# The FACT Histone Chaperone: Tuning Gene Transcription in the Chromatin Context to Modulate Plant Growth and Development

**DOI:** 10.3389/fpls.2020.00085

**Published:** 2020-02-19

**Authors:** Klaus D. Grasser

**Affiliations:** Cell Biology & Plant Biochemistry, Biochemistry Centre, University of Regensburg, Regensburg, Germany

**Keywords:** SSRP1, Pob3, SPT16, histone chaperone, *Arabidopsis*, chromatin

## Abstract

FACT is a heterodimeric histone chaperone consisting of the SSRP1 and SPT16 proteins and is conserved among eukaryotes. It interacts with the histones H2A-H2B and H3-H4 as well as with DNA. Based on *in vitro* and *in vivo* studies mainly in yeast and mammalian cells, FACT can mediate nucleosome disassembly and reassembly and thus facilitates in the chromatin context DNA-dependent processes including transcription, replication and repair. In plants, primarily the role of FACT related to RNA polymerase II transcription has been examined. FACT was found to associate with elongating *Arabidopsis* RNA polymerase II (RNAPII) as part of the transcript elongation complex and it was identified as repressor of aberrant intragenic transcriptional initiation. *Arabidopsis* mutants depleted in FACT subunits exhibit various defects in vegetative and reproductive development. Strikingly, FACT modulates important developmental transitions by promoting expression of key repressors of these processes. Thus, FACT facilitates expression of *DOG1* and *FLC* adjusting the switch from seed dormancy to germination and from vegetative to reproductive development, respectively. In the central cell of the female gametophyte, FACT can facilitate DNA demethylation especially within heterochromatin, and thereby contributes to gene imprinting during *Arabidopsis* reproduction. This review discusses results particularly from the plant perspective about the contribution of FACT to processes that involve reorganisation of nucleosomes with a main focus on RNAPII transcription and its implications for diverse areas of plant biology.

## Chaperoning Histones

In eukaryotes, the nuclear DNA is packaged into nucleosomes, which represent the basic repeat unit of chromatin. The nucleosome contains 147 bp of DNA wrapped around an octamer composed of two copies each of the four core histones H2A, H2B, H3, and H4. Adjacent nucleosomes are connected by linker DNA of variable length (10–80 bp depending on cell type and species) that typically associates with linker histones such as H1 (McGhee and Felsenfeld, 1980; Luger and Richmond, 1998; Kornberg and Lorch, 1999). The general stability of nucleosome particles imposes major obstacles to transcription and other DNA-dependent processes (Li et al., 2007). Therefore, different mechanisms have evolved that facilitate chromatin transcription by destabilising/disassembly of nucleomes (Henikoff, 2008; Zhou et al., 2019). In the regulation of nucleosome dynamics, in addition to other factors so-called histone chaperones are critically involved. Histone chaperones are a heterogeneous class of proteins that functionally interact with core histones to assemble/disassemble nucleosome particles without consuming energy in form of ATP and they are not necessarily part of the final product (De Koning et al., 2007). There are various types of histone chaperones that contribute to different chromatin-related processes including transcription, replication, and DNA repair (Das et al., 2010; Avvakumov et al., 2011; Gurard-Levin et al., 2014; Hammond et al., 2017). Often histone chaperones are classified as either H2A-H2B or H3-H4 chaperones, reflecting their preferential interaction with different core histones. Some histone chaperones even display selectivity towards specific H3 or H2A isoforms such as replicative or replacement variants (De Koning et al., 2007; Hammond et al., 2017). Beyond that histone chaperones have been functionally linked with the occurrence of certain post-translational modifications of core histones and thus with the establishment, maintenance and propagation of specific chromatin states (Avvakumov et al., 2011).

Due to the extensive evolutionary conservation of the structure of the nucleosome particle, many of the histone chaperones that have been studied in detail in yeast and metazoa also occur in plants. Thus, a variety of H2A-H2B and H3-H4 chaperones have been identified throughout the plant kingdom (Tripathi et al., 2015; Zhou et al., 2015; Kumar and Vasudevan, 2020). By modulating local chromatin structure histone chaperones were found to contribute to the regulation of plant growth and development (Ramirez-Parra and Gutierrez, 2007; Otero et al., 2014; Takatsuka and Umeda, 2015; Zhou et al., 2015). Moreover, tuning of chromatin states by histone chaperones to mediate altered gene expression programs can assist plants to cope more efficiently with environmental stress conditions (Zhu et al., 2013; Liu et al., 2015; Probst and Mittelsten Scheid, 2015).

In this article, the current knowledge about the histone chaperone FACT will be summarised, particularly its role in *Arabidopsis*, as most studies in plants were performed using this model. At first, though the discovery of FACT and its mode of action in yeast and metazoa is introduced.

## Discovery of FACT and Its Molecular Role in Chromatin Transactions

Originally, FACT (FAcilitates Chromatin Transcription) was identified in yeast and mammalian cells (Brewster et al., 1998; Orphanides et al., 1998; Orphanides et al., 1999; Wittmeyer et al., 1999). Its name originates from the finding that FACT promoted *in vitro* transcription from reconstituted chromatin templates by destabilising nucleosomes, facilitating RNA polymerase II passage during elongation (Orphanides et al., 1998; Orphanides et al., 1999; Belotserkovskaya et al., 2003). Over the years it became clear that besides chromatin transcription, FACT is also involved in other chromatin-dependent processes such as replication, recombination, and repair (Belotserkovskaya et al., 2004; Singer and Johnston, 2004; Winkler and Luger, 2011; Formosa, 2012; Gurova et al., 2018), and hence, the established name may well stand more broadly for facilitates chromatin transactions. FACT is a heterodimer consisting of the SSRP1 (Structure-Specific Recognition Protein 1; termed Pob3 in yeast) and SPT16 (SuPpressor of Ty 16). The main feature of FACT is its ability to disassemble and reassemble nucleosomes, and thus its involvement in overcoming and maintaining the nucleosomal barrier to DNA-dependent processes occurring in the chromatin context. Accordingly, FACT can interact with various nucleosomal targets including H2A-H2B dimers, H3-H4 tetramers and DNA (Jamai et al., 2009; Winkler et al., 2011; Hondele et al., 2013; Kemble et al., 2015). The nature of FACT-histone interactions has been further elucidated in a recent cryo-EM study of human FACT in complex with partially assembled sub-nucleosomes (Liu et al., 2020). This work illustrates that structure of FACT resembles a unicycle, consisting of a saddle and fork that is engaged in extensive interactions of SSRP1 and SPT16 with nucleosomal DNA and all histones. Competition between FACT and DNA for histone binding seems to be critical for reversible nucleosome reorganisation and uncoiling of the nucleosomal DNA from the histone core that generally leads to increased DNA accessibility (Xin et al., 2009; Hondele et al., 2013; Kemble et al., 2015; Tsunaka et al., 2016; Valieva et al., 2016; Wang et al., 2018). Following transient nucleosome destabilisation, for instance, during passage of transcribing RNA polymerase II, FACT promotes nucleosome reassembly that is important to maintain proper chromatin signature and to prevent aberrant transcriptional initiation from cryptic promoters (Kaplan et al., 2003; Mason and Struhl, 2003; Cheung et al., 2008; Jamai et al., 2009; Wang et al., 2018). Further intriguing molecular and structural details of numerous studies on yeast and metazoan FACT are summarised in various excellent review articles (Belotserkovskaya et al., 2004; Winkler and Luger, 2011; Formosa, 2012; Gurova et al., 2018).

## Basic Facts About Plant FACT

**Figure 1:**
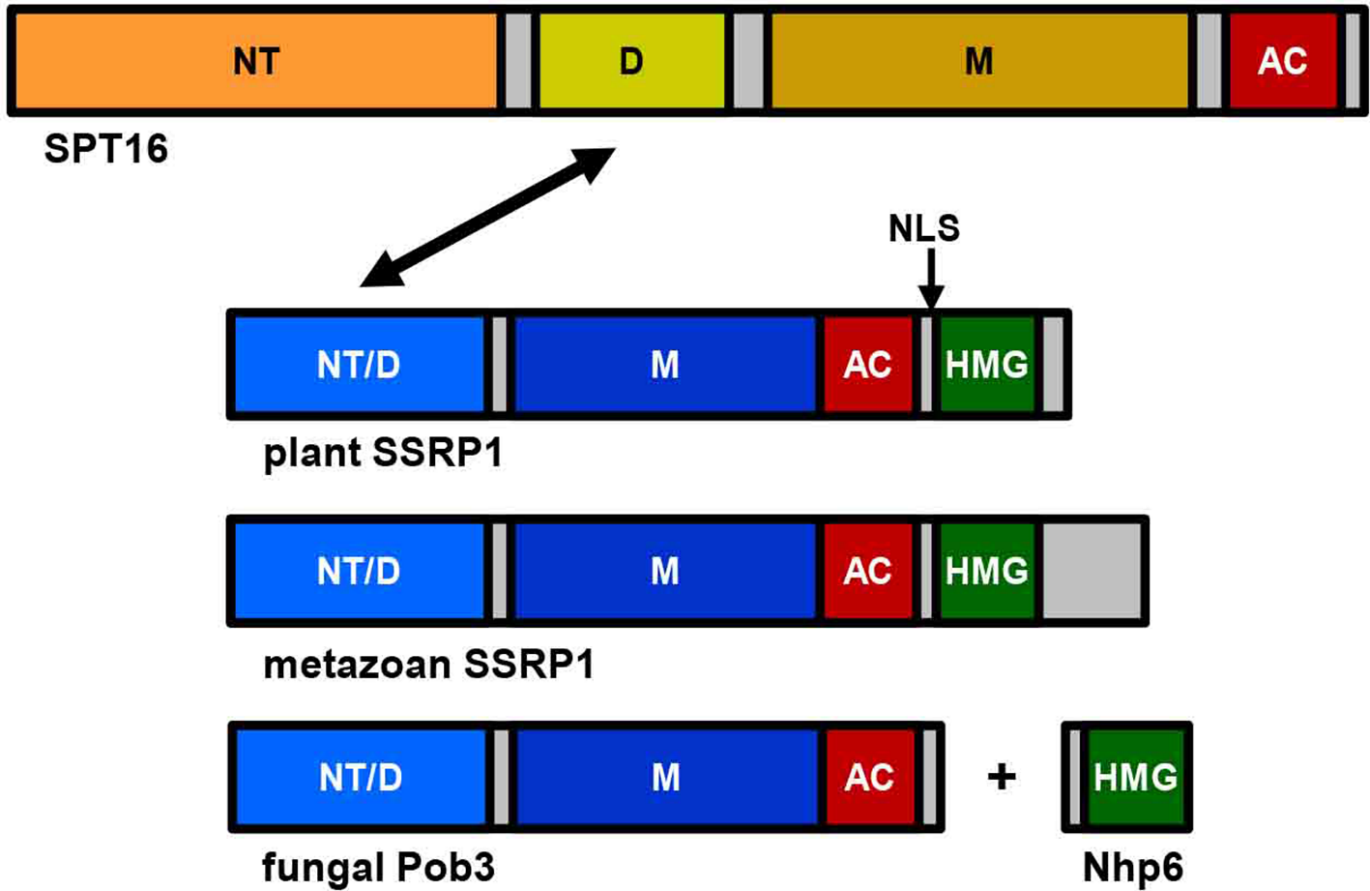
Schematic representation of the FAcilitates Chromatin Transcription (FACT) subunits SPT16 and SSRP1. While the overall structure of SPT16 is essentially conserved throughout eukaryotes, there are differences in the C-terminal region of SSRP1 (Pob3 in fungi). SPT16 consists of N-terminal domain (NT), dimerisation domain (D), middle domain (M), and acidic C-terminal domain (AC), while SSRP1/Pob3 proteins of different eukaryotes are composed of N-terminal domain (that is required for heterodimerisation (NT/D) with SPT16, indicated by an arrow), middle domain (M), acidic region (AC), and HMG-box domain (HMG), which in case of yeast Pob3 is provided by the separate protein(s) Nhp6a/b. Plant SSRP1 contains a nuclear localisation signal (NLS, indicated by an arrow) within a short basic region linking the acidic domain and the HMG-box (Röttgers et al., 2000).

**Figure 2:**
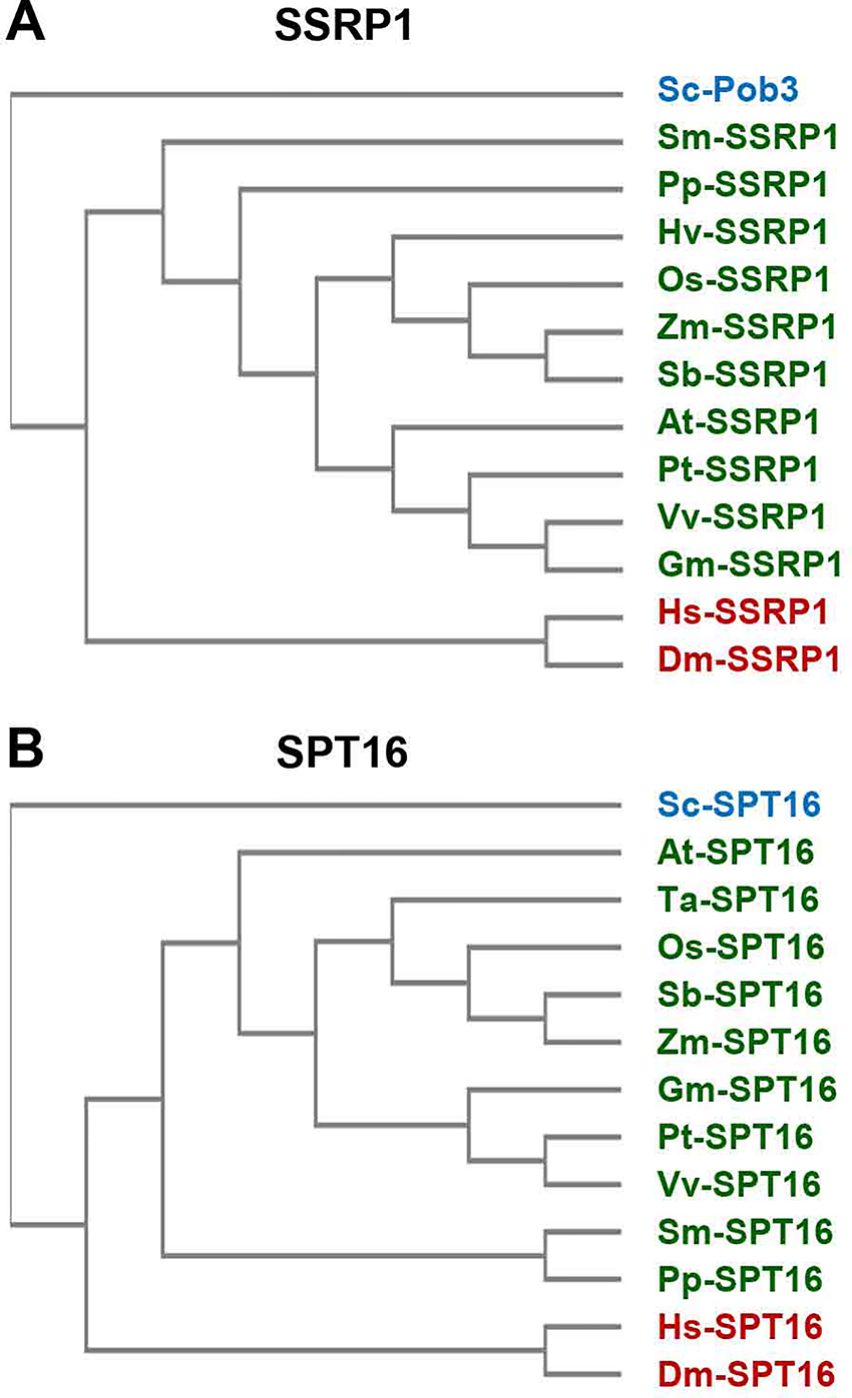
Sequence similarity of FAcilitates Chromatin Transcription (FACT) subunits. The amino acid sequences of SSRP1 **(A)** and SPT16 **(B)** proteins from various organisms (*Arabidopsis thaliana (At), Drosophila melanogaster (Dm), Glycine max (Gm), Homo sapiens (Hs), Hordeum vulgare (Hv), Oryza sativa (Os), Physcomitrella patens (Pp), Populus trichocarpa (Pt), Saccharomyces cerevisiae (Sc), Selaginella moellendorfii (Sm), Sorghum bicolor (Sb), Triticum aestivum (Ta), Vitis vinifera (Vv), Zea mays (Zm))* were aligned by multiple sequence alignment (cf. **Supplementary Figures S1**, **S2**) using Clustal Omega (https://www.ebi.ac.uk/Tools/msa/clustalo/) that served to cluster the sequences (unweighted pair group method with arithmetic mean). The sequences of plants are indicated in green and those of metazoa in red, while the yeast sequence is labelled blue.

The FACT heterodimer consisting of SSRP1 (71.6 kDa) and SPT16 (120.6 kDa) was demonstrated by reciprocal coimmunoprecipitation from *Arabidopsis* cells (Duroux et al., 2004). SPT16 comprises an N-terminal domain, a dimerisation domain, a middle domain, and an acidic C-terminal domain (**Figure 1**), and the overall domain organisation of plant SPT16 closely resembles the counterparts of other eukaryotes (**Supplementary Figure S1**). SSRP1 contains an N-terminal domain that mediates dimerisation with SPT16, a middle domain, an acidic domain, and a C-terminal HMG-box domain (**Figure 1**). Metazoan SSRP1 differs from the plant orthologues by a more pronounced C-terminal extension, while the fungal orthologues lack the HMG-box domain (**Supplementary Figure S2**) that in yeast is provided by separate small HMGB-box proteins termed Nhp6a/b (Formosa, 2012; Gurova et al., 2018). Proteins closely related to *Arabidopsis* SSRP1 and SPT16 are encoded by monocot and dicot plants, as well as by *Selaginella* and *Physcomitrella* (**Figure 2**).

Since SSRP1 contains an HMG-box domain that typically mediates DNA-interactions (Antosch et al., 2012; Malarkey and Churchill, 2012), the DNA-binding properties of maize SSRP1 were examined. These experiments revealed that SSRP1 does not interact with DNA sequence-specifically, but according to a binding-site selection assay, it binds preferentially to sequences containing deformable dinucleotide steps (Röttgers et al., 2000). In line with this finding, mediated by its HMG-box domain SSRP1 can bend linear DNA and bind selectively to certain DNA structures (Röttgers et al., 2000; Pfab et al., 2018b). Furthermore, SSRP1 is phosphorylated by protein kinase CK2 and phosphorylation of two residues C-terminal of the HMG-box domain modulates the structure-specific interaction with DNA (Krohn et al., 2003). The HMG-box domain of SSRP1 is not only important for DNA-binding, but contributes also to nucleosome interactions (Lichota and Grasser, 2001; Pfab et al., 2018b). In view of the relevance of the HMG-box domain for *in vitro* DNA/nucleosome interactions, it was surprising that based on fluorescence recovery after photobleaching experiments intact SSRP1 and SSRP1 lacking its HMG-box domain (termed SSRP1∆HMG) displayed the same mobility within nuclei of *Arabidopsis* cells. Beyond that, expression of SSRP1∆HMG was almost as efficient as that of intact SSRP1 in supporting normal growth and development of the otherwise nonviable *ssrp1-1* mutant (Pfab et al., 2018b). This suggested that the HMG-box domain, which is conserved among SSRP1 proteins of plants and metazoa, is not critical in *Arabidopsis* under standard growth conditions. Possibly, FACT containing SSRP1∆HMG (or intact SSRP1) functionally interacts with small abundant HMGB proteins similar to the mechanism reported for yeast FACT. Yeast Pob3 lacks the C-terminal HMG-box domain (thus structurally resembling *Arabidopsis* SSRP1∆HMG; cf. **Figure 1**) and the HMG-box function is provided by small Nhp6a/b HMG-box proteins (Brewster et al., 2001; Formosa et al., 2001). However, fusing a C-terminal HMG-box domain to Pob3 is insufficient for full, Nhp6-independent activity. Both yeast FACT containing the Pob3-HMG fusion and human FACT were dependent on the presence of Nhp6 for efficient nucleosome reorganisation (McCullough et al., 2018). Collectively, these findings suggest that SSRP1-SPT16 of plants/metazoa may need assistance of small HMGB proteins in a way analogous to the cooperation of Pob3/SPT16 with Nhp6 in yeast. However, this issue requires further investigations.

Both SSRP1 and SPT16 are nuclear proteins and are ubiquitously expressed in all/most *Arabidopsis* tissues, but expression is not detectable in certain terminally differentiated cells such as mature trichoblasts or cells of the root cap (Duroux et al., 2004; Ikeda et al., 2011; Pfab et al., 2018a). Consistent with the enrichment of SSRP1 in the highly micrococcal nuclease-sensitive fraction of chromatin (Lichota and Grasser, 2001), both SSRP1 and SPT16 are detected by indirect immunofluorescence microscopy in the euchromatin of interphase nuclei, but not in heterochromatic chromocenters (Duroux et al., 2004). Using chromatin immunoprecipitation SSRP1-SPT16 was detected along the transcribed region of genes transcribed by RNAPII, but not at loci transcribed by RNA polymerases I and III or intergenic regions. Moreover, association with the chromatin of active protein-coding genes occurred in a transcription-dependent manner (Duroux et al., 2004; Perales and Más, 2007; Lolas et al., 2010; Antosz et al., 2017). In accordance with that an affinity-purification approach combined with mass spectrometry demonstrated that FACT efficiently copurified with elongating RNAPII (CTD-phosphorylated at residues S2P, S5P) from *Arabidopsis* cells as well as with known transcript elongation factors including TFIIS, SPT4/SPT5 and PAF1C (Antosz et al., 2017). Moreover, *SSRP1* and *SPT16* genetically interact with *TFIIS* encoding a modulator of RNAPII activity and with *HUB1/2*, encoding factors catalysing elongation-related mono-ubiquitination of histone H2B (Lolas et al., 2010; Antosz et al., 2017). Taken together these findings indicate a role of *Arabidopsis* FACT in RNAPII transcriptional elongation (Van Lijsebettens and Grasser, 2014; Zhou et al., 2015; Grasser and Grasser, 2018), in line with the function of FACT as regulator of mRNA synthesis in other organisms (Reinberg and Sims, 2006; Formosa, 2012; Gurova et al., 2018), although the exact mechanism *in vivo* is still obscure.

Intriguing insight provided a study analysing genome-wide intragenic transcriptional initiation from cryptic promoters in *Arabidopsis*. Thousands of discrete, mostly exonic transcriptional start site positions were mapped in *ssrp1* and *spt16* mutants and the majority of these sites were detected in both mutants (Nielsen et al., 2019). This suggested that FACT is required for repression of aberrant intragenic transcript initiation, whereas no evidence was found for an involvement in repression of cryptic transcription by other elongation factors such as PAF1C, Elongator and the SDG8 H3K36-methyltransferase. At FACT-repressed intragenic start sites an enrichment of the RNAPII elongation signature H3K4me1 was detected that may contribute to suppress intragenic transcriptional initiation (Nielsen et al., 2019). Since FACT has been implicated in repressing cryptic transcription also in other organisms (Kaplan et al., 2003; Mason and Struhl, 2003; Cheung et al., 2008; Jamai et al., 2009), ensuring transcriptional fidelity by restricting transcript initiation to promoters may be a key function of FACT.

## FACT in Plant Growth and Development

**Figure 3:**
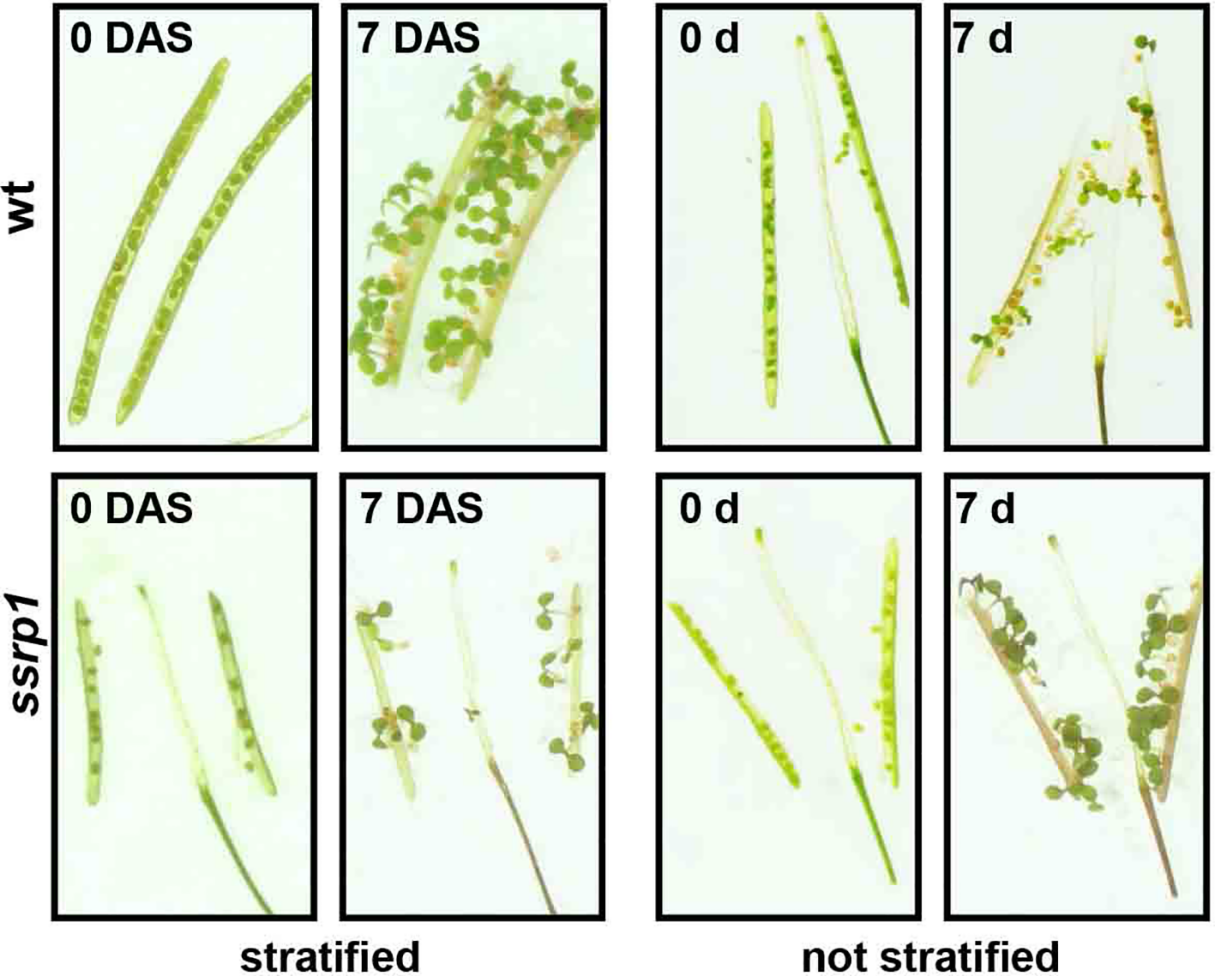
Reduced dormancy of *ssrp1* seeds. Germination assays with opened siliques harvested 14 days after flowering. They are shown at day 0 and 7 days after incubation, either with or without prior stratification. Note the smaller siliques of *ssrp1* containing a severely reduced number of seeds compared to wt. After stratification almost all seeds germinate, whereas without stratification wt seeds germinate inefficiently (< 50%), whereas *ssrp1* seeds due to reduced seed dormancy germinate efficiently (~90%).

In various organisms including *Arabidopsis*, FACT is essential for viability (Cao et al., 2003; Lolas et al., 2010; Formosa, 2012; Frost et al., 2018). *Arabidopsis* mutant plants expressing reduced amounts of SSRP1 or SPT16 similarly show various defects in vegetative and reproductive development. Thus, the mutant plants display an increased number of leaves and inflorescences as well as altered leaf architecture (Lolas et al., 2010). In addition, these plants are early bolting, have abnormal flower morphology and a severely reduced seed set. The early bolting phenotype is associated with reduced expression of the floral repressor *FLC* in *ssrp1* and *spt16* plants relative to the wild type controls (Lolas et al., 2010). Germination assays with freshly harvested seeds demonstrated that in contrast to the wild type control, *ssrp1* mutant seeds germinated efficiently without stratification (**Figure 3**), indicating reduced seed dormancy (Michl-Holzinger et al., 2019). In line with this phenotype, *ssrp1* seeds exhibit decreased transcript levels of the *DOG1* gene, which is a known quantitative trait locus of seed dormancy. Introduction of an additional copy of *DOG1* into *ssrp1* resulted in increased *DOG1* transcript levels and consistently in more robust seed dormancy (Michl-Holzinger et al., 2019). Therefore, SSRP1 is required for efficient expression of *DOG1* and FACT is a modulator of seed dormancy in *Arabidopsis*. These findings reveal that FACT is involved in two of the most important plant developmental switches, namely, the transition from seed dormancy to germination and from vegetative to reproductive development. Interestingly, both processes in addition to FACT are regulated by other modulators of transcriptional elongation and chromatin structure. Thus, factors including FACT, PAF1C, SWR1, SDG8 and HUB1/2 contribute to the expression of *FLC* in the induction to flowering (He et al., 2004; Oh et al., 2004; Zhao et al., 2005; Choi et al., 2007; Cao et al., 2008; Lázaro et al., 2008; Lolas et al., 2010), while factors such as FACT, TFIIS, H2B-monoubiquitinases, and H3-methylases influence the expression of *DOG1* in the switch from seed dormancy to germination (Liu et al., 2007; Zheng et al., 2012; Molitor et al., 2014; Michl-Holzinger et al., 2019). Furthermore, FACT was identified as cofactor of the transcriptional regulation of circadian rhythms in *Arabidopsis*. Initially, it was observed that FACT rhythmically associates with the circadian oscillator gene *TOC1* (Perales and Más, 2007). Subsequently, protein interactions were detected between FACT, elongating RNAPII and clock-related components termed LNKs. By interaction between LNKs and the MYB factor RVE8 the transcription machinery is recruited to target promoters, leading to rhythmic occupancy of clock gene promoters (Ma et al., 2018). FACT could be involved in this scenario facilitating the transition from RNAPII transcript initiation to productive elongation.

Recent transcript profiling of 10-day-old *Arabidopsis ssrp1* and *spt16* seedlings in comparison to the wild type demonstrated that a relatively small subset of genes is differentially expressed in the mutants (Pfab et al., 2018a). The alterations in the transcript profile of both mutants relative to wild type were very similar, consistent with the function of SSRP1 and SPT16 as a heterodimer. Among the downregulated genes, those encoding enzymes of anthocyanin biosynthesis were remarkably overrepresented. Upon exposure to moderate high-light stress several of the anthocyanin biosynthetic genes were induced in the *ssrp1/spt16* plants to a lesser extent than in the wild type, and accordingly the mutant plants depleted in FACT accumulated lower amounts of anthocyanin pigments. Expression of SSRP1 and SPT16 was increased under these conditions (Pfab et al., 2018a). Therefore, FACT is required for transcriptional induction leading to anthocyanin accumulation in response to light stress.

A special role of FACT that was discovered in *Arabidopsis* is its involvement in parent-of-origin specific gene expression (genomic imprinting). Initially, SSRP1 was found to be required for DNA demethylation and activation/repression of parentally imprinted genes in the central cell of the female gametophyte (Ikeda et al., 2011). The authors proposed that SSRP1 might contribute to altering the chromatin state, facilitating demethylation by the DNA demethylase DEMETER (DME). Subsequently, bimolecular complementation assays indicated that SSRP1 and SPT16 colocalised with DME in the nucleus. Genome-wide analyses demonstrated that SSRP1 and SPT16 are required for demethylation at over half the DME-mediated demethylation sites in the central cell (Frost et al., 2018). DME demethylation sites that are particularly dependent on FACT occur in heterochromatic domains with high nucleosome occupancy and are enriched in H3K9me2 and H3K27me1 marks, whereas euchromatic DME targets apparently can be demethylated by the enzyme without assistance of FACT (Frost et al., 2018). Therefore, FACT may be required for DME targeting by facilitating its access to heterochromatic sites, but the exact molecular role of FACT in this process is unknown. Moreover, the authors suggest that the mode of FACT action in conjunction with DME during reproduction differs from that during transcriptional elongation.

## Perspectives

There is substantial evidence that FACT in yeast and metazoa is involved in addition to transcription in various other DNA-dependent processes including replication, recombination and repair. To date essentially the role of plant FACT in transcription has been addressed, and therefore, broader approaches are required to gain insight to which extent it contributes to additional biologically crucial processes in plants. Open questions regarding FACT include how it is recruited to its target sites in chromatin. Analyses in yeast, for instance, indicate that FACT associates with the transcribed regions of all active RNAPII-transcribed genes (Mayer et al., 2010). However, various studies suggest that the absence of FACT causes rather moderate changes in gene expression of relatively small subsets of genes (Gurova et al., 2018). This raises the question, of why the transcription of certain genes is more dependent on FACT than the majority of other genes. Which gene characteristics determine the requirement for FACT action? There exist many potentially influencing parameters including DNA sequence, chromatin structural features, inducibility and expression level of the gene, RNAPII density and elongation rate, as well as cotranscriptional mRNA processing. Perhaps a combination of these and additional parameters defines the requirement of FACT for efficient transcription. Finally, because of the various functions of FACT in nucleosome reorganisation in different biological contexts it appears likely that FACT activity is regulated, but currently this is largely obscure. Although many facts about FACT have been elucidated in recent years, there remain important open questions.

## Author Contributions

KG wrote the manuscript.

## Funding

German Research Foundation (DFG) through grants SFB960/A6 and Gr1159/14-2.

## Conflict of Interest

The author declares that the research was conducted in the absence of any commercial or financial relationships that could be construed as a potential conflict of interest.
