# Supplementary material for: The FACT Histone Chaperone: Tuning Gene Transcription in the Chromatin Context to Modulate Plant Growth and Development
Source: Front Plant Sci. 2020 Feb 19;11:85. doi: 10.3389/fpls.2020.00085 (PMC7042381; doi:10.3389/fpls.2020.00085)
Supplement: Supplementary file 1 [file DataSheet_1.pdf]

```
Sc-Pob3       --MSTDFDRIYLN--QSKFSGRFRIADSGLGWKISTSGGSAANQARKPFLLPATELSTVQ   56
Sm-SSRP1      MGDAHQFNNILLGGRGGANPGQLKISSKGFSWRKSGGGKV--------VDVAKDDVKSVS   52
Pp-SSRP1      ----HQFNSILLGGRGGTNPGQLKINSGGFQWRKAGGGKV--------VEVSTSDIASLS   48
Hv-SSRP1      MTDGHHFNNILLGGRAGTNLGQFKVHSGGLAWKRQGGGKT--------IEIDKADLTSVT   52
Os-SSRP1      MTDGHLFNNILLGGRAGSNPGQFKVYSGGLAWKRQGGGKT--------IEIEKSDLTSVT   52
Zm-SSRP1      MTDGHHFNNILLGGRGGTNPGQFKVHSGGLAWKRQGGGKT--------IEIDKADVTAVT   52
Sb-SSRP1      MTGGHHFNNILLGGRGGTNPGQFKVYSGGLAWKRQGGGKT--------IEIDKADVTAVT   52
At-SSRP1      MADGHSFNNISLSGRGGKNPGLLKINSGGIQWKKQGGGKA--------VEVDRSDIVSVS   52
Pt-SSRP1      MTDGHLFNNIFLGGRGGTNPGHLKISPGGILWKKQGGGKA--------VEVDRADILGVT   52
Vv-SSRP1      MSEGHLFNNISLGGRGGTNPGQLRVHPGGILWKKQGGGKA--------VEVDKSDIVGVT   52
Gm-SSRP1      MTDGHLFNNITLGGRGGTNPGQIKIYPGGIIWKRQGGGKL--------IEVDKSDIMGVT   52
Dm-SSRP1      MTDSLEYNDINAEVRGVLCSGRLKMTEQNIIFKNTKTGKV--------EQISAEDIDLIN   52
Hs-SSRP1      MAETLEFNDVYQEVKGSMNDGRLRLSRQGIIFKNSKTGKV--------DNIQAGELTEGI   52
                    :: :          * :::   .: ::    *            :   ::

Sc-Pob3       WSRGCRGYDLKINTKNQGVIQLDGFSQDDYNLIKNDFHRRFNIQVEQREHSLRGWNWGKT   116
Sm-SSRP1      WTRVSRSYQMSIRLKAGSNVKFTGFREQDVSSLNTFITSNIGVTPQDKPLAVSGRNWGEA   112
Pp-SSRP1      WTRVPKGFQLGVKLKAGRDVKFNGFRESDMANLGSFFTTNMGITSEEKPLAISGRNWGDV   108
Hv-SSRP1      WMKVPRAYQLGVRVKDGLSYTFIGFREQDVSSLINFMQKNMGISPDEKQLSVGGHNWGMI   112
Os-SSRP1      WMKVPRAYQLGVRTKDGLFYKFIGFREQDVSSLTNFMQKNMGLSPDEKQLSVSGQNWGGI   112
Zm-SSRP1      WMKVPRAYQLGVRIKAGLFYRFIGFREQDVSNLTNFIQKNMGVTPDEKQLSVSGQNWGGI   112
Sb-SSRP1      WMKVPRAYQLGVRIKDGLFYRFIGFREQDVSSLTNFIQKNMGVTPDEKQLSVSGHNWGGI   112
At-SSRP1      WTKVTKSNQLGVKTKDGLYYKFVGFRDQDVPSLSSFFQSSYGKTPDEKQLSVSGRNWGEV   112
Pt-SSRP1      WMKVPRTNQLSVLIKGGPWYKFTGFRDQDLSTLTNFFQ-SHGITPEEKQLSVSGRNWGEV   111
Vv-SSRP1      WMKVPRTNQLGVRVKDGLYYKFTGFREQDVTNLTNFFQHSCGLNPEEKQLSVSGRNWGEV   112
Gm-SSRP1      WMKVPRSNQLGVQIKDGLYYKFTGFRDQDVVSLTNFFQNTCGISVEEKQLSVSGRNWGEV   112
Dm-SSRP1      SQKFVGTWGLRVFTKGGVLHRFTGFRDSEHEKLGKFIKAAYSQEMVEKEMCVKGWNWGTA   112
Hs-SSRP1      WRRVALGHGLKLLTKNGHVYKYDGFRESEFEKLSDFFKTHYRLELMEKDLCVKGWNWGTV   112
                :      : :  *        ** :.:   :   :         ::  .: * ***

Sc-Pob3       DLARNEMVFALNGKPTFEIPYARINNTNLTSKNEVGIEFNIQDEEYQP---AGDELVEMR   173
Sm-SSRP1      ELEGNTLSFVVGGKPAFEICVADISNTQVQGKNDVQMEFHVDD-----TANEKDTLMEMS   167
Pp-SSRP1      TLEGSSLAFSVGGKHAFDVSIADVSQTQMQGKNEVMLEFHVDD---TTGASEKDTLMELS   165
Hv-SSRP1      DIDGSMLTFMVGSKQAFEFSLADVSQTQLQGKTDVLLEFHVDD---TTGANEKDSLMDMS   169
Os-SSRP1      DINGNMLTFMVGSKQAFEVSLADVSQTQMQGKTDVLLEFHVDD---TTGGNEKDSLMDLS   169
Zm-SSRP1      DIDGNMLTFMVGSKQAFEVSLPDVAQTQMQGKTDVLLELHVDD---TTGANEKDSLMDLS   169
Sb-SSRP1      DIDGNMLTFMVGSKQAFEVSLADVAQTQMQGKTDVLLELHVDD---TTGANEKDSLMDLS   169
At-SSRP1      DLHGNTLTFLVGSKQAFEVSLADVSQTQLQGKNDVTLEFHVDD---TAGANEKDSLMEIS   169
Pt-SSRP1      DLNGNMLTFLVGSKQAFEVSLADVSQTQMQGKNDVILEIDAIISNYILLCLQKDSLMELS   171
Vv-SSRP1      DLNGNMLTFLVGSKQAFEVSLADVSQTQMQGKNDVILEFHVDD---TTGANEKDSLMEIS   169
Gm-SSRP1      DLNGNMLAFTVGSKQAFEVSLADVSQTQLQGKNDVILEFHVDD---TTGANEKDSLMEIS   169
Dm-SSRP1      RFMGSVLSFDKESKTIFEVPLSHVSQC-VTGKNEVTLEFHQND-------DAPVGLLEMR   164
Hs-SSRP1      KFGGQLLSFDIGDQPVFEIPLSNVSQC-TTGKNEVTLEFHQND-------DAEVSLMEVR   164
               :  . : *   .:  *:.    : :    .*.:* :*:.               *:::

Sc-Pob3       FYIPGVIQTNVDENMTKKEESSNEVVPKKEDGAEGEDVQMAVEEKSMAEAFYEELKEKAD   233
Sm-SSRP1      FHVPSTNTTFVG-----------------------------DEERASAQVFCDKILSMAD   198
Pp-SSRP1      FHIPTTNTTYIG-----------------------------DEERPPAQIFREKILSMGD   196
Hv-SSRP1      FHVPTSNTQFPG-----------------------------NENRTSAQILLEAILERAD   200
Os-SSRP1      FHVPTSNTQFLG-----------------------------DENRTAAQVLWETIMGVAD   200
Zm-SSRP1      FHVPTSNTQFVG-----------------------------DESRPPAHILWETILKFAD   200
Sb-SSRP1      FHVPTSNTQFVG-----------------------------DENRPPAHILWETILKFAD   200
At-SSRP1      FHIPNSNTQFVG-----------------------------DENRPPSQVFNDTIVAMAD   200
Pt-SSRP1      FHIPNNNTQYIG-----------------------------DENHPPAQVFRDLIVQKAD   202
Vv-SSRP1      FHIPNSNTQFVG-----------------------------DENRPPAQVFRDKIMSMAD   200
Gm-SSRP1      FHIPNSNTQFVG-----------------------------DENRPPAQVFRDKIMSMAD   200
Dm-SSRP1      FHIPAVES----------------------------------AEEDPVDKFHQNVMSKAS   190
Hs-SSRP1      FYVPPTQE----------------------------------DGVDPVEAFAQNVLSKAD   190
              *::*                                            . : : :   ..

Sc-Pob3       IGEV-AGDAIVSFQDVFFTTPRGRYDIDIYKNSIRLRGKTYEYKLQHRQIQRIVSLPKAD   292
Sm-SSRP1      VGPA-GGEAVAAFETVNVLTPRGRYLVELHLSFLRLFG-ASDFKIQYTSVARLFVLPRSA   256
Pp-SSRP1      VGPS-GAEAIALFEEVHILTPRGRYNVELHASFLRLQGQANDFKIQYSSVVRLFILPKSN   255
Hv-SSRP1      TSSGSSGEAVVTFEGIAILTPRGRYAVDLHLSFLRLQGQANDFKIQYSSIVRLFLLPKSN   260
Os-SSRP1      V--DSSEEAVVTFEGIAILTPRGRYSVELHLSFLRLQGQANDFKIQYSSIVRLFLLPKSN   258
Zm-SSRP1      V--GSSEEPVVTFEGIAILTPRGRYSVELHLSFLRLQGQANDFKIQYSSIVRLFLLPKSN   258
Sb-SSRP1      V--GSSEEPVVTFDGIAILTPRGRYSVELHLSFLRLQGQANDFKIQYSSIVRLFLLPKSN   258
At-SSRP1      VSPG-VEDAVVTFESIAILTPRGRYNVELHLSFLRLQGQANDFKIQYSSVVRLFLLPKSN   259
Pt-SSRP1      VGAG-GEEAVVTFEGIAILTPRGRYSVELHLSFLRLQGQANDFKIQYSSVVRLFLLPKFN   261
Vv-SSRP1      VGAG-GEEAVVTFEGIAILTPRGRYSVELHLSFLRLQGQANDFKIQYSSVVRLFLLPKSN   259
Gm-SSRP1      VGAG-GEDAIVTFEGIAILTPRGRYSVELHMSFLRLQGQANDFKIQYSSVVRLFLLPKSN   259
Dm-SSRP1      VISA-SGESIAIFREIQILTPRGRYDIKIFSTFFQLHGKTFDYKIPMDSVLRLFMLPHKD   249
Hs-SSRP1      VIQA-TGDAICIFRELQCLTPRGRYDIRIYPTFLHLHGKTFDYKIPYTTVLRLFLLPHKD   249
                     : :  *  :   ****** : :. . ::* * : ::*:    : *:. **:
```

```
Sc-Pob3     DIHHLLVLAIEPPLRQGQTTYPFLVLQFQKDEETEVQLNLEDEDYEENYKDKLKKQYDAK  352
Sm-SSRP1    QSSTYVVITLDPPIRKGQTFYPHIVFQYPSEEITELTPLISDELLNTKYKDRLAPQYKGL  316
Pp-SSRP1    QPHTFVVITLDPPIRKGQTFYPHIVLQFPSEEIAECTLSIGEELLNTKYKDRLQPAYKDL  315
Hv-SSRP1    NPHTIVVVTLDPPIRKGQTLYPHIVIQFETETVVQKNMKLSRELLDEKYKDRLEESYQGL  320
Os-SSRP1    NPHTFVVVTLDPPIRKGQTLYPHIVIQFETEAVVERNLALTKEVLAEKYKDRLEESYKGL  318
Zm-SSRP1    NPHTFVVITLDPPIRKGQTLYPHIVIQFETEAVVERDLALSKELLVEKYKDRLEESYKGL  318
Sb-SSRP1    NPHTFVVITLDPPIRKGQTLYPHIVIQFETEAVVERDLALSKELLVDKYKDRLEESYKGL  318
At-SSRP1    QPHTFVVISLDPPIRKGQTMYPHIVMQFETDTVVESELSISDELMNTKFKDKLERSYKGL  319
Pt-SSRP1    QPHTFVVVTLDPPIRKGQTLYPHIVLQFDTDFVVQSNLSMSEDLLYTKYKDKLEPSYKGL  321
Vv-SSRP1    QPHTFVVVTLDPPIRKGQTLYPHIVMQFETDYVVQSELSLSEELLNSKYKDKLEPSYKGL  319
Gm-SSRP1    QPHTFVIISLDPPIRKGQTLYPHIVMQFETDYVVESELAINEDLYNTKYKDKLDLSYKGL  319
Dm-SSRP1    SRQMFFVLSLDPPIKQGQTRYHYLVLLFAPDEETTIELPFSEAELRDKYEGKLEKEISGP  309
Hs-SSRP1    QRQMFFVISLDPPIKQGQTRYHFLILLFSKDEDISLTLNMNEEEVEKRFEGRLTKNMSGS  309
            .    .:::::**:::*** * .::: :  :        :       .::.:*    .

Sc-Pob3     THIVLSHVLKGLTDRRVIVPG-EYKSKYDQCAVSCSFKANEGYLYPLDNAFFFLTKPTLY  411
Sm-SSRP1    SYEVFVEILRGLSGAGITKPGTKFRSSQEGYAVRAALKAEEGLLYPLEKSFFFLPKPPTL  376
Pp-SSRP1    SHDVFTQILKGLSGAKVTRPG-KFRSAQDGYCVRTSLKAEEGTLYPLEKSFFFLPKPPTL  374
Hv-SSRP1    VHEVFVKVLRGLSGAKVTRPG-SFRSYKNGYAVKSSLKAEDGLLYPLEKGFFFLPKPPTL  379
Os-SSRP1    IHEVFTKVLRGLSGAKVTRPG-SFRSCQDGYAVKSSLKAEDGLLYPLEKGFFFLPKPPTL  377
Zm-SSRP1    IHEVFTKVLRGLSGAKVTRPG-SFRSCQDGYAVKSSLKAEDGLLYPLEKGFFFLPKPPTL  377
Sb-SSRP1    IHEVFTKVLRGLSGAKVTRPG-SFRSCQDGYAVKSSLKAEDGLLYPLEKGFFFLPKPPTL  377
At-SSRP1    IHEVFTTVLRWLSGAKITKPG-KFRSSQDGFAVKSSLKAEDGVLYPLEKGFFFLPKPPTL  378
Pt-SSRP1    IHEVFTTILRGLSSAKVTRPG-KFRSCQDGYAVKSSLKAEDGVLYPLEKSFFFLPKPPTL  380
Vv-SSRP1    IHEVFTLILRGLSGAKVTKPG-KFRSCQDGYAVKSSLKAEDGVLYPLEKSFFFLPKPPTL  378
Gm-SSRP1    IHEVFTTILRGLSGAKVTKPG-KFRSCQDGYAVKSSLKAEDGILYPLEKSFFFLPKPPTL  378
Dm-SSRP1    VYEVMGKVMKVLIGRKITGPG-NFIGHSGTAAVGCSFKAAAGYLYPLERGFIYIHKPPLH  368
Hs-SSRP1    LYEMVSRVMKALVNRKITVPG-NFQGHSGAQCITCSYKASSGLLYPLERGFIYVHKPPVH  368
             : :.  ::: * .  :  ** .: .     .:  : **  * ****:..*::: **

Sc-Pob3     IPFSDVSMVNISRAGQTS---TSSRTFDLEVVLRSNRGSTTFANISKEEQQLLEQFLKSK  468
Sm-SSRP1    IVYEEIDYVEFERHGAAGTSSMSSKNFDLILRLKTD-QEHLFRNISRNEYHSLFKFISEK  435
Pp-SSRP1    ILHDEIEYLEFERHGAAGTSSISSHYFDLLIRLKSE-QEHQFRNIQRNEYHNLFNFISGK  433
Hv-SSRP1    ILHEEIEFVEFQRHGAGG-ASMSSQYFDLLVKLTND-QEHLFRNIQRSEYHNLFNFINGK  437
Os-SSRP1    ILHEEIEFVEFERHGAGG-ASISSHYFDLLVKLKND-QEHLFRNIQRSEYHNLFNFINGK  435
Zm-SSRP1    ILHEEIEFVEFERHGAGG-ASISSHYFDLLVKLKND-QEHLFRNIQRNEYHNLFNFINGK  435
Sb-SSRP1    ILHEEIEFVEFERHGAGG-ASISFHYFDLLVKLKND-QEHLFRNIQRNEYHNLFNFINGK  435
At-SSRP1    ILHDEIDYVEFERHAAGG-ANM--HYFDLLIRLKTD-HEHLFRNIQRNEYHNLYTFISSK  434
Pt-SSRP1    ILHEEIDYVEFERHAAGG-SNM--HYFDLLIRLKTE-QEHLFRNIQRNEYHNLFDFISGK  436
Vv-SSRP1    ILHEEIDYVEFERHAAGG-SNM--HYFDLLIRLKTE-QEHLFRNIQRNEYHNLFDFISGK  434
Gm-SSRP1    ILHEEIDYVEFERHAAGG-SNM--HYFDLLIRLKSE-QEHLFRNIQRNEYHNLYEFISSK  434
Dm-SSRP1    IRFEEISSVNFARSGGST------RSFDFEVTLKNG-TVHIFSSIEKEEYAKLFDYITQK  421
Hs-SSRP1    IRFDEISFVNFARGTTTT------RSFDFEIETKQG-TQYTFSSIEREEYGKLFDFVNAK  421
            * ..::. ::: *           : **: :          * .*.:.*   *  ::. *

Sc-Pob3     NLRVKNEDREVQE---RLQTALGSDSDEEDIN--MG---------S------AGEDDESV  508
Sm-SSRP1    GLKILNFQTQG---SSAVAEALQ-GSDDEGVDPHLERIRIARATG-------DEGDSDEE  484
Pp-SSRP1    NLKIMNLGDAQG--TSGVAAALE-GSDDEGVDPHLNRIRSARESGGAGL---GDEDSDEE  487
Hv-SSRP1    GLKVMNLG-DGQGTS-GVTDVLR-DTDDVAPDPHLERIKNQA------V---SSEASDEE  485
Os-SSRP1    HLKIMNLG-DGQGATGGVTAVLR-DTDDDAVDPHLERIKNQA------G---D-EESDEE  483
Zm-SSRP1    NIKIMNLGGDGQGASGVVTDVLR-DTDDDAVDPHLERIKNQA------G---D-EESDEE  484
Sb-SSRP1    NIKIMNLGGDGQGASGVVTDVLR-DTDDDAVDPHLERIKNQA------G---D-DESDEE  484
At-SSRP1    GLKIMNLGGAG-T-ADGVAAVLGDNDDDDAVDPHLTRIRNQA------A-----DESDEE  481
Pt-SSRP1    GMKIMNLGDMQ-T-AKGVAAVLQ-NDDDDAVDPHLARIRNEA------G---D-DESDEE  483
Vv-SSRP1    GLKIMNLGDVQ-T-ADGVAAVLQ-NDDDDAVDPHLERIKNEA------G---G-DESDEE  481
Gm-SSRP1    GLKILNLGDAQ-P-TVGIKKVLE-NDDDDAVDPHLERIKNEA------G---G-DESDEE  481
Dm-SSRP1    KLHVSNMGKDKSGY---KDVDFGDSDNENEPDAYLARLKAEAREKEEDDD-DGDSDEEST  477
Hs-SSRP1    KLNIKNRGLKEGMN---PSYDEYADSDEDQHDAYLERMKEEGKIREENANDSSDDSGEET  478
             :.: *                  . ::   :  :                      :.

Sc-Pob3     DEDFQVSSDNDADEVAEEFDSDAA--------LSDAEGGSDEERPSKKPKVE--------  552
Sm-SSRP1    DEDFVADK---DDEGSPTDDSGEE----EEPDASDSGGEQQDERPVKKDKKKVEASQK--  535
Pp-SSRP1    DEDFVAEK---DDAGSPTDESDGE-----EPDGSDDGGERPKKKREKREDVAPKSA----  535
Hv-SSRP1    DEDFVLHK---DDGGSPTDDSGGE-----ESDASESGG--EKEKSSKKEARSSKPPVKRK  535
Os-SSRP1    DEDFVADK---DDSGSPTDDSGGE-----DSDASESGG--EKEKLSKKEASSSKPPVKRK  533
Zm-SSRP1    DEDFVADK---DDSGSPTDDSGDE-----ESDASDSGG--EKEKSSKKEASSSKPVQKRK  534
Sb-SSRP1    DEDFVADK---DDSGSPTDDSGDE-----DSDASDSGG--EKEKSSKKEASSSKPVQKRK  534
At-SSRP1    DEDFVMGE--DDDGGSPTDDSGGD-----DSDASEGGVGEIKEKSIKKEPKKEASSS---  531
Pt-SSRP1    DEDFVLGK---DDGGSPTDDSGEE-----ESDASESGD--EKENPGKKDFKREVSSS---  530
Vv-SSRP1    DEDFVLDK---DDGGSPTDDSGEE-----ESDASESGG--EKEKPSKKESKKEPSVS---  528
Gm-SSRP1    DSDFVADK---DDEGSPTDDSGAD-----DSDATDSGD--EKEKPAKKESKKDLPSK---  528
Dm-SSRP1    DEDFKPNE--NESDVAEEYDSNVESDSDDDSDASGGGG--DSDGAKKKKEKKSEKKE---  530
Hs-SSRP1    DESFNPGE--EEEDVAEEFDSNASASSSSN------EG--DSDRDEKKRKQL--------  520
            *..*   .    .  :   :*.                   ..   *:
```

```
Sc-Pob3       ------------------------------------------------------------   552
Sm-SSRP1      P------A-ATKKKKKDEEGDGDKKKKRRKKDPNAPKRALSGFMYFSLAERENLKKS--T   586
Pp-SSRP1      P------KKRKKKDEADGEDGGKKKRKKKEKDPNAPKRALSAFMRFQLEERKKMKADPGQ   589
Hv-SSRP1      PKGKDGEGSEKRKPKGKDGEGSEKRKPKKKKDPNAPKRPMMPFMYFSMAERAGVKDS--N   593
Os-SSRP1      PKGRDE-------------EGSDKRKPKKKKDPNAPKRAMTPFMYFSMAERGNMKNN--N   578
Zm-SSRP1      HKARDD-------------EGQEKKKPKKKKDPNAPKRAMTPFMYFSMAERGNMKSS--N   579
Sb-SSRP1      PKARDD-------------EGHEKKKAKKKKDPNAPKRAMTPFMYFSMAERGNMKSS--N   579
At-SSRP1      -----KGLPPKRKTVAADEGSSKRKKPKKKKDPNAPKRAMSGFMFFSQMERDNIKKE--H   584
Pt-SSRP1      -----KAVTKRKS--RDGEESQKKRKPKKKKDPNAPKRSKSAYVFFSQMERENVKKS--N   581
Vv-SSRP1      -----KASSSKKKPKDGDEDGSKKRKQKKKKDPNAPKRAMSGFMFFSQTERENIKKS--T   581
Gm-SSRP1      ------ASTSKKK--SKDDEDGKKRKQKKRKDPNAPKRAMSGFMFFSKLERENLKKT--N   578
Dm-SSRP1      ---------KKEKKHKEKERT--KKPSKKKKDSGKPKRATTAFMLWLNDTRESIKRE--N   577
Hs-SSRP1      ---------KKAKMAKDRKSRKKPVEVKKGKDPNAPKRPMSAYMLWLNASREKIKSD--H   569

Sc-Pob3       ------------------------------------------------------------   552
Sm-SSRP1      PGISFKDVAKTLGERWKAMSKDEKEPFESQARVDKERYTKQMQGYNKGNAGGSTADDESS   646
Pp-SSRP1      ASMSFGEFGKSLGEKWRNMSASDKAPYEADAKVDQERYKKAMAGYK--------------   635
Hv-SSRP1      PDLAPTDVAKKLGEMWQKMSTEDKQPYILQSQADKKRYEKESAAYRA--AAPVDVDAGSG   651
Os-SSRP1      PDLPTTEIAKKLGEMWQKMTGEEKQPYIQQSQVDKKRYEKESAVYRG--AAAMDVDSGSG   636
Zm-SSRP1      PDLPTTEIAKKLGEMWQKMSGEEKQPYIQQAQVDKKRYEKESAVYRG--EATVDVDSGNE   637
Sb-SSRP1      PDLPTTEIAKKLGEMWQKMSSEEKQPYIQQAQVDKKRYEKESAVYRG--EATADVDSGNE   637
At-SSRP1      PGIAFGEVGKVLGDKWRQMSADDKEPYEAKAQVDKQRYKDEISDYK--NPQPMNVDSGND   642
Pt-SSRP1      PGIVFGEITKALADKWNAMSAEEKEPYEEMARDDKQRYKSQVNDYKNKNPQPMMVDSGYE   641
Vv-SSRP1      PGIAFTEVGRVLGDKWKKMTAEEKEPYEAKAQADKKRYRDEISGYK-SNPQPMNVDSGNE   640
Gm-SSRP1      PGISFTDVSRVLGEKWKKLSVEEKEPYEAKAREDKKRYKDEISGYK--NPQPMNIDSGNE   636
Dm-SSRP1      PGIKVTEIAKKGGEMWKELKD--KSKWEDAAAKDKQRYHDEMRNYKPEAGGDSDNEKGGK   635
Hs-SSRP1      PGISITDLSKKAGEIWKGMSKEKKEEWDRKAEDARRDYEKAMKEYEGGRGESSKRDKSKK   629

Sc-Pob3       ------------------------------------------------------------   552
Sm-SSRP1      D-----------------------------------------------------------   647
Pp-SSRP1      ------------------------------------------------------------   635
Hv-SSRP1      NGSD--------------------------------------------------------   655
Os-SSRP1      GNESD-------------------------------------------------------   641
Zm-SSRP1      SD----------------------------------------------------------   639
Sb-SSRP1      SD----------------------------------------------------------   639
At-SSRP1      SDSN--------------------------------------------------------   646
Pt-SSRP1      SDS---------------------------------------------------------   644
Vv-SSRP1      SDSE--------------------------------------------------------   644
Gm-SSRP1      SDSA--------------------------------------------------------   640
Dm-SSRP1      SSKK-----RKTEPSPS----KKANTSGSGFKSKEYISDDDSTSSDDEKDNEPAKKKSKP   686
Hs-SSRP1      KKKVKVKMEKKSTPSRGSSSKSSSRQLSESFKSKEFVSSDESSSGENKSKKKRR-----R   684

Sc-Pob3       -------------------------------------         552
Sm-SSRP1      -------------------------------------         647
Pp-SSRP1      -------------------------------------         635
Hv-SSRP1      -------------------------------------         655
Os-SSRP1      -------------------------------------         641
Zm-SSRP1      -------------------------------------         639
Sb-SSRP1      -------------------------------------         639
At-SSRP1      -------------------------------------         646
Pt-SSRP1      -------------------------------------         644
Vv-SSRP1      -------------------------------------         644
Gm-SSRP1      -------------------------------------         640
Dm-SSRP1      PSDGDAKKKKAKSESEPEESEEDSNASDEDEEDEASD         723
Hs-SSRP1      SEDSE----EELASTPPSSEDSASGSDE---------         709
```

Figure S1. Amino acid sequence alignment of SSRP1 from different organisms. Sequences (plant, green; metazoan, red; fungal, blue) were retrieved by database searches using blastp (https://blast.ncbi.nlm.nih.gov/) using At-SSRP1 as query. The sequences were aligned using Clustal Omega (https://www.ebi.ac.uk/Tools/msa/clustalo/). The HMG-box domain present in SSRP1 proteins is highlighted in yellow, but it is absent from Sc-Pob3 (and other fungal Pob3 sequences; Pfab et al., 2018). Compared to their plant counterparts, metazoan SSRP1 proteins have a more extended domain C-terminal of the HMG-box. The species of which the protein sequences were retrieved are indicated as follows: *Arabidopsis thaliana (At), Drosophila melanogaster (Dm), Glycine max (Gm), Homo sapiens (Hs), Hordeum vulgare (Hv), Oryza sativa (Os), Physcomitrella patens (Pp), Populus trichocarpa (Pt), Saccharomyces cerevisiae (Sc), Selaginella moellendorfii (Sm), Sorghum bicolor (Sb), Vitis vinifera (Vv), Zea mays (Zm).*
