# Supplementary material for: The FACT Histone Chaperone: Tuning Gene Transcription in the Chromatin Context to Modulate Plant Growth and Development
Source: Front Plant Sci. 2020 Feb 19;11:85. doi: 10.3389/fpls.2020.00085 (PMC7042381; doi:10.3389/fpls.2020.00085)
Supplement: Supplementary file 2 [file DataSheet_2.pdf]

```
Sc-SPT16      ------------------------------MEELNIDFDVFKKRIELLYSKYNEFEGS--   28
At-SPT16      ----------MADSRNGNARAPPSGVPPKAGNTYSIDVKNFISRARALYEHWKKHSA---   47
Ta-SPT16      ------------MTDNGKAK-SG------SGAAYTINLETFSKRLKVFYDHWNGNKS---   38
Os-SPT16      ------------MADNGNAK-PGG----GGSGAYTINLDNFSKRLKVFYDHWKEHNS---   40
Sb-SPT16      ------------MADNGNA----K----GGSGAYTINLENFSKRLKVFYDHWKEHKS---   37
Zm-Spt16      MVALVNVSLLTNMADNGDA----K----GGSGAYAINIENFSKRLKVFYDHWKEHKS---   49
Gm-SPT16      ----------MADHRNGSAQ-AANGKASAAGTAYSIDLNAFQTRLRSFYKHWDAHKT---   46
Pt-SPT16      ----------MADQRNGTGQ-PSN----AARNAYAIDVEKFKTRLKALYSNWNENKA---   42
Vv-SPT16      ----------MAEHRNGNAK-PSDGKASGAASPYAINLDNFTKRLKTLYSHWKEHSS---   46
Sm-SPT16      ----------MADSRNGGDA-----KSKANDSGYDINIDDFVKRLNIFYKCWADEKN---   42
Pp-SPT16      ----------MAE-RNG----------DGKGGRVQINLELYGKRLKLLNNKWKEHKK---   36
Hs-SPT16      -------------------------------MAVTLDKDAYYRRVKRLYSNWRKG----E   25
Dm-SPT16      ------------------------------MSSFVLDKEAFVRRVKRLYTEWRAPSIGHD   30
                                                 :: . :  * . :   :

Sc-SPT16      -----PNSLLFVL--GSSNAENPYQKTTILHNWLLSYEFPATLIALVPGKVIIITSSAKA   81
At-SPT16      DLWGSADALAIATPPAS--DDLRYLKSSALNIWLLGYEFPDTIMVFTKKQIHFLCSRNKA   105
Ta-SPT16      DLWASSDAIAIATPPPS--EDLRYLKSTALDVWLLGYEFPETIIVFMQKQIHFLCSQKKA   96
Os-SPT16      DLWGSSNAIAIATPPPS--EDLRYLKSSALDVWLLGYEFPETIIVFMHKQIHFLCSQKKA   98
Sb-SPT16      DLWSSSDAIAIATPPPS--DDLRYLKSSALDIWLLGYEFPETIIVFMHKQIHVLCSQKKA   95
Zm-Spt16      DLWGSSDAIAIATPPPS--DDLRYLKSSALDIWLLGYEFPETIIVFMHKQIHVLSSQKKA   107
Gm-SPT16      DLWGSSDAIAVACPPPS--EDLRYLKSTALNLWLLGFEFPETIMVFSKKQIHILCSQKKA   104
Pt-SPT16      DLWGSSDVVAIATPPPS--EDLRYLKSSALNIWLLGYEFPETVMVFMKKQIHFLCSQKKA   100
Vv-SPT16      DLWGSSDALAIATPPAS--DDLRYLKSSALNIWLLGYEFPETIMVFMKKQIHFLCSQKKA   104
Sm-SPT16      ELWGGADAVAVFTPPRAEASELRYLKSSALNIWMLGYEFPDTLMVFVQGALHFLCSQKKV   102
Pp-SPT16      EMWGGADAIAVVTPPAS--EDLRYLKSTALHIWLLGYEFPETVMVFMPGALHFVCSSKKA   94
Hs-SPT16      DEYANVDAIVVS---VGVDEEIVYAKSTALQTWLFGYELTDTIMVFCDDKIIFMASKKKV   82
Dm-SPT16      DALRNLDCIMSI---VGVEEDVMYSKSMALQLWLLGYELTDTISVFCSDAVYFLTSKKKI   87
                    : :       .   :  * *:  *. *::.:*:  *: .:    : .: *  *

Sc-SPT16      KHLQKAIDLFK----DPESKITLELWQRNNKEPELNKKLFDDVIALIN---SAGKTVGIP   134
At-SPT16      SLLEVVKKPA---HDELKLDVIMHVKPKGDDGTGLMDAIFRAIRDLSRGDGNDSQVVGHI   162
Ta-SPT16      NLIGTLKDAA---SEAVGSDIILHVKSKNGDGIDLMDDILRAVSAQSK---SDTPVVGHI   150
Os-SPT16      NLIGTLKKAA---NDAVGADIVLHVKAKNDSGVGLMEDIVRAVCAQSK---SDDPIVGHI   152
Sb-SPT16      NLIGTLKKAA---NESVGVDIVLHVKTKNGDGADLMDHIVQAARNQSK---SDKPVVGHI   149
Zm-Spt16      NLIGTLKKAA---NEAVGVDIVLHVKTKNSDGADLMDDIVHAARNQSK---SDKPVVGHI   161
Gm-SPT16      SILESVKKTA---REAVDADLVLHVKPRNDDGTALMDAIFRALS---KSDGRDTPTVGYI   158
Pt-SPT16      SLLEVVKKPA---REVVGVDVVMHVKAKTDNGTGLMDAIFHAIYAQSSADGKDTPVVGHI   157
Vv-SPT16      SLLEVVRKSA---KEAVGVEVVMHVKAKSDDGTGLMDAIFRAVRANSS--SHDTPVVGHI   159
Sm-SPT16      KILEELQRPA---RTSCGVDVVLHVKLRSEDGGPQMLEILDTVKAQ-----SRSPVLGVL   154
Pp-SPT16      AHLEELQKSS---KMLTGVDIHIHMKERKQDGSVQMNSVLDAVKGFSK---GKTPTVGVL   148
Hs-SPT16      EFLKQIANTKGNENANGAPAITLLIREKNESNKSSFDKMIEAIKES-----KNGKKIGVF   137
Dm-SPT16      EFLKQTQNI----TEEGFPEINLLVRDRTDKDQGNFEKLIKALQNS-----KKGKRLGVF   138
                :                 : : :  :  .       :.                :*

Sc-SPT16      EKDSYQGKFMTEWNPVWEAAVKENEFNVIDISLGLSKVWEVKDVNEQAFLSVSS-KGSDK   193
At-SPT16      AREAPEGKLLETWTE----RLKNANFQFVDITGGLSDLFAVKDDTEVMSVKKAAYLAYSV   218
Ta-SPT16      AKEAPEGKLLETWAE----KLAGGSVQLADVTHGFSELFAVKDATEIICVKKAAYLTSSV   206
Os-SPT16      AKEAPEGKLLEAWAD----KLSSSSVQLTDITNGFSELFAMKDTSEITCVKKASYLTSSV   208
Sb-SPT16      AKEVPEGKLLETWTE----KLSGSGVRLTDVTNGFSELFAVKDTTEVTCVKKAAYLTSSV   205
Zm-Spt16      AKEAPEGKLLETWIK----KLSGSGLRLVDVTNGFSELFAVKDTTEITCVKKAAYLTSSV   217
Gm-SPT16      SREAPEGKLLEMWTE----KLKNTKFQLNDVANGLSSLFAAKNNEELTSIKRAAYLTTSV   214
Pt-SPT16      AREAPEGIILETWAE----KLKGEGFELADVTSGLSDLIAVKDADELINVKKAAFLTFSV   213
Vv-SPT16      GREAPEGKLLEMWTE----KLKNADFQLSDITNGFSDLFAMKDSTELTNVKKAAFLTSSV   215
Sm-SPT16      AKEKTEGSFMEKWDE----LLSSSRLEKVDVAAGLCEMFAVKDESEINNVKKAAYLSATA   210
Pp-SPT16      SREATEGSVMEKWAE----CLEASGAATVDVSGGFSEIFAVKDEEEISNIKNASHLSAAI   204
Hs-SPT16      SKDKFPGEFMKSWND----CLNKEGFDKIDISAVVAYTIAVKEDGELNLMKKAASITSEV   193
Dm-SPT16      AKDAYPGEFSEAWKK----SLTASKFEHVDISTIIAYLMCPKDESEINNIRKASLVSMDI   194
               ::   * .   *       :        *::  ..     *:  *   :  ::

Sc-SPT16      FMDLLSNEMVRAVDEELKITNAKLSDKIENKIDDVKFLKQLSPDLSALCPPNYKFNFDLL   253
At-SPT16      MKNVVVPNLESAIDEEKDVTHSALMDLTEKAILEPTK------------ASVKLKPENV   265
Ta-SPT16      MKNFVVPTMEKVIDEERKVSHSSLMDDTEKVILDPLK------------AKVKLKPENI   253
Os-SPT16      MKNFVVPKLEKVIDEERKVTHSSLMDETEKAILDPLK------------VKVKLKAENV   255
Sb-SPT16      LKNFVVPKLEKVIDEEKKVSHSSLMDDTEKAILDPLK------------VKVKLKPDNV   252
Zm-Spt16      LKNFVIPKLEKVIDEEKEVSHSSLMDDAEKAILDPLK------------VKVKLKPDNV   264
Gm-SPT16      MKNFVVPKLENVIDEEKKVSHSTLMEDTEKAILEPSK------------VNCKLKADNV   261
Pt-SPT16      MNNVVVPKLENVIDEEKNITHSALMDEAEKAILDPTR------------AKAKLKADNV   260
Vv-SPT16      MKHFVVPKLEKVIDEEKKVSHSSLMDDTEKAILEPAR------------VKVKLKAENV   262
Sm-SPT16      MKTFVVPKLEHVIDEEKRMTHAELMEETENVMLDPPGK-----------LKMKLKAEGA   258
Pp-SPT16      LKSFVVPKLEVIIDEEKKVTHFELMEQTENVITNPGKY-------------VKLKAEDV   250
Hs-SPT16      FNKFFKERVMEIVDADEKVRHSKLAESVEKAIEEKK-------------YLAGADPSTV   239
Dm-SPT16      FNKYLKDEIMDIIDSDRKVKHNKLSDGCEAAIGEKK-------------YTSGLDPRLL   240
              :   .   :   :* :  : :  *  :  *  : :                     .
```

```
Sc-SPT16      DWTYSPIIQSGKKFDLRVSARSTNDQLYGN--GCILASCGIRYNNYCSNITRTFLIDPSE  311
At-SPT16      DICYPPIFQSGGKFDLKPSAASNDELLTYDPASIIICAVGARYNSYCSNVARTYLIDATS  325
Ta-SPT16      DICYPPVFQSGGKFDLRPGASSNDDYLYYDPASIIICAIGSRYSNYCSNVARTFLIDATP  313
Os-SPT16      DICYPPVFQSGGKFDLKPGASSNDDYLYYDSASVIICAIGARYGNYCSNMARTFLIDATP  315
Sb-SPT16      DICYPPVFQSGGKFDLKPGASSNDEYLYYDSASVIICAIGSKYSSYCSNVARTYLIDATP  312
Zm-Spt16      DICYPPVFQSGGKFDLKPGASSNDEYLYYDSASIIICAIGSKYSSYCSNVARTYLIDATP  324
Gm-SPT16      DICYPPIFQSGGEFDLKPSAVSNDELLHYDSASVILCAIGARYKSYCSNIARTFLIDADP  321
Pt-SPT16      DICYPPIFQSGGEFDLRPSAASNDEPLYYDSASVIIIAVGSRYNSYCSNVARTLMIDATP  320
Vv-SPT16      DICYPPIFQSGGEFDLRPSASSNDENLYYDSTSVIICAIGSRYNSYCSNVARTFLIDANA  322
Sm-SPT16      DVCYPPIFQSGGVFDLKASAQSNEDPLYYDATAVIICALGGRYNMYCSNVARTYLIDADT  318
Pp-SPT16      DICYPPVFQSGGVFDLKPSAVSNEEPLYYDTLGVILCAIGARFRSYCSNVARSIMIDADK  310
Hs-SPT16      EMCYPPIIQSGGNYNLKFSVVSDKNHMHF---GAITCAMGIRFKSYCSNLVRTLMVDPSQ  296
Dm-SPT16      DMAYPPIIQSGGAYSLKFSAVADKNPLHF---GVIVCSLGARYKSYCSNISRTFLVNPTE  297
              :  * *::***  :.*: .. : .: :     . *  : * ::  ****: *: :::

Sc-SPT16      EMANNYDFLLTLQKEIVTNILKPGRTPKEVYESVIEYIEKTKPELVPNFTKNIGSLIGLE  371
At-SPT16      LQSKAYEVLLKAHEAAI-DALRSGRKINTVYQAALSVVEKNAPEFVDKLTKSAGTGIGLE  384
Ta-SPT16      AQSKAYETLLKAQEAAL-AACKPGNQMSAVFKAAVAVFEKNAPELLPNLTKSAGTGIGLE  372
Os-SPT16      TQIKAYETLMKAHEAAL-EALKPGNRMSAVYQAAVDVIEKNAPELLRNLTKSAGTGIGLE  374
Sb-SPT16      KQSKAYETLLKAHEAAV-QEVKPGNQMSAVYQAAVAVIEKDAPELLPNLTKSAGTGIGLE  371
Zm-Spt16      TQNKAYETLRKAHEAAI-QQVKPGNQMSAVYQAAVAVIERDAPELLPNLTKSAGTGIGLE  383
Gm-SPT16      LQSRAYGVLLKAHEAVI-GSLKPGNRLSATYQAAVSVVENEAPDLISYLTKSAGTGIGIE  380
Pt-SPT16      LQSKAYAVLLKAHEAAI-GALKPGNKVSAAYQAALSVVEEEAPELVPNLSKSAGTGIGLE  379
Vv-SPT16      MQSKAYEVLLKAHEAAI-GALKPGNKVSAAYQAALAVVEKDAPELVSNLTKSAGTGIGLE  381
Sm-SPT16      SQEKAYKALLKAQEAAI-NALRPGNLMSAVYKAAATTLEREAPELSSFLTRSAGAGIGIE  377
Pp-SPT16      TQEKAYKILLKAHEAAI-AALRPGNAMSVAYKAAYAVVESGGPEFLPYFTKNAGTGIGIE  369
Hs-SPT16      EVQENYNFLLQLQEELL-KELRHGVKICDVYNAVMDVVKKQKPELLNKITKNLGFGMGIE  355
Dm-SPT16      AMQENYTFLVSVQEEIL-KLLVPGTKLCDVYEKTLDFVKKEKPSMVDNLPKSFGFAMGLE  356
                 . *  *   ::  :       *     .:: .   .:   *.:   : :. *  :*:*

Sc-SPT16      FRDSNFILNVKNDYRKIQRGDCFNISFGFNNLKDSQS----ANNYALQLADTVQIPLDET  427
At-SPT16      FRESGLNINAKNDK-VLRPKMAFNVSLGFQNLECESESRSKNKKFSLLLADTVLVTDQ--  441
Ta-SPT16      FRESGLNLNAKNDR-LIKEGMIFNVNLGLSNIQAETN-NEKTKQFSLLLADTALVNDK--  428
Os-SPT16      FRESGLNLNPKNDR-IIKAGMVFNVSLGLHNLQAEKK-SEKTKQYSLLLADTCLVPL---  429
Sb-SPT16      FRESGLNLNAKNDR-KIKQGMVFNVSLGLHNVQAETT-SEKTKQFSLLLADTVLVNER--  427
Zm-Spt16      FRESGLNLNAKNDR-RIKKGMVFNVSLGLHNIQAETT-SEKTKQFSLLLADTVLVNER--  439
Gm-SPT16      FRESGLNINAKNEQ-IVKEGMVFNVSLGFQNVQREST-KSKSKHFSLLLADTVIINKD--  436
Pt-SPT16      FRESGLNLNAKNDR-VVKAKMVFNVSLGFQNLQNQID-NPKIRNFSLLLADTVIVGDQ--  435
Vv-SPT16      FRESGLNLNAKNDR-VLKPGMVFNVSLGFQNLQTDTN-NPKTQKFSVLLADSVIVGEK--  437
Sm-SPT16      YRESGLSLNPRNEK-VIKAGMVFNVNIGLQNLEIKSS-NPKTRTYGLLLADTIVVRDK--  433
Pp-SPT16      FRESGLTLNAKNER-VIRPGMAFNVSLGFHNLTTESS-NPKSKTFSLLLADTAIVVEK--  425
Hs-SPT16      FREGSLVINSKNQY-KLKKGMVFSINLGFSDLTNKEGKKPEEKTYALFIGDTVLVDED--  412
Dm-SPT16      FRENSIVIGPKCQA-LLKKNMVFNLHVGISNLTNPEATDKEGKNYALFIGDTVLVGEQ--  413
              :*:..: :. : :   ::    *.: .*: ::           . :.: :.*:  :

Sc-SPT16      EPPRFLT-NYTKAKSQISFYFNNEEEDNNKKKS-SPATKVPSKPDRNSKILRTKL-RG-E  483
At-SPT16      KPELL-T-KCSKSVKDVAYSFKEDEE---E---EKPRK----KARTSG-SE-NYITKTAL  487
Ta-SPT16      AAEIL-T-NCSKAVKDVAYSFNEDEEEVPK----PKRA----KVEPNG-VE-ALPSKATL  476
Os-SPT16      --ENLTA-SCSKLVKDVAYSFNDEDEVLPV----KK------VEVNAK-EA-LPPTKATL  474
Sb-SPT16      GHDILTA-PCSKAVKDVAYSFNEDDEDV-A----EV------KMESKT-ID-VMPTKATL  473
Zm-Spt16      GHEVLTA-PCSKAFKDVAYSFNEDDDAVAA----EV------KIKSKT-ID-VMPTKATL  486
Gm-SPT16      KTEVVTS-MSSKALKDIAYSFNEDEE---E---ENPSA----KADANG-AE-PLMSKTTL  483
Pt-SPT16      NPDVVTS-KSSKAVKDVAYSFNEGEE--EE---QKPKA----RAEVNG-GE-NLMSKTTL  483
Vv-SPT16      GPEVVTS-ISSKAVKDVAYSFNEDDDEEEE---ERPKV----KPEANG-GE-AVSSKATL  487
Sm-SPT16      GPDVVTS-LSSKAFQDIAYSFKDGDEEPEERPRS--------KPASNG-AE-PVYVKTAL  482
Pp-SPT16      GPPEVPTLKCSKTYTDIAYSFKDDEEDEEVKVEAKPKV----KSESNGSNE-PAVRMATL  480
Hs-SPT16      GPATVLT-SVKKKVKNVGIFLKNEDEEEEEEE-----------KDEAEDLLGRGSRAALL  460
Dm-SPT16      SPASVMT-PSKKKIKNVGIFIKDDSDEEDVDDK--KTA----KEDQGTEILGRSKRNAVL  466
                  . :   .*   ::.  ::: .:

Sc-SPT16      ARGGAEDAQKEQIRKENQKKLHEKLEKNGLLRFSAAD-ANGPDSEPRQYFKKYESYVRDS  542
At-SPT16      -RSDDHVVSKEELRKQHQAELARQKNEETARRLAGDSSGAGDSRSTAKTSADVVAYKNVN  546
Ta-SPT16      -RSDNQEMSKEELRRQHQAELARQKNEETARRLAGGGSGNGDGRGPSRNSNELVAYKNVN  535
Os-SPT16      -RSDNQEMSKEELRRQHQAELARQKNEETARRLAGVGSGSGDGRGPSRSSNELVAYKNVN  533
Sb-SPT16      -RSDNQEMSKEELRRQHQAELARQKNEETARRLAGVGSGSGDGRGPARASNELVAYKNVN  532
Zm-Spt16      -RSDNQEMSKEELRRQHQAELARQKNEETARRLAGVGTGSGDGRGPARASNELVAYKNVN  545
Gm-SPT16      -RSDNHEMSKEELRRQHQAELARQKNEETARRLAGGGNETGDNRSSSRTSAELVAYKNIN  542
Pt-SPT16      -RSDNGEISKEELRRQHQAELARQKNEETARRLAGGGSAKGDNRAASKTSTDLVAYKNVN  542
Vv-SPT16      -RSDNQEMSKEELRRQHQAELARQKNEETARRLAGGGSGAGDNRGAVKATGDLIAYKNVN  546
Sm-SPT16      -RSDNQEMTKEDQRRQMQAELALKKNEETARRLAAGAFGHGEGHNMVKSSGEMTAYRNVD  541
Pp-SPT16      -RSDNQEMTKEEQRRQHQAELARQKNEETARRLASGGLGSGDGQGPNKTTGDIIAYRNVD  539
Hs-SPT16      TERTRNEMTAEEKRRAHQKELAAQLNEEAKRRLTEQKGE----QQIQKARKSNVSYKNPS  516
Dm-SPT16      ESKLRNEINTEEKRKEHQRELAQQLNERAKDRLARQGNS----KEVEKVRKNTVSYKSIS  522
                        *: *:  * :*  : ::.   *::              :   .  :*   .
```

```
Sc-SPT16      QLP--TNIRDLRIHVDWKSQTIILPIYGRPVPFHINSYKNGSKNEEG-EYTYLRLNFNSP   599
At-SPT16      DM---PH-KELMIQVDTRNEAVLLPIYGSLVPFHVATIRTVSGNQDTNRNCYIRIIFNVP   602
Ta-SPT16      DV---PYSRELVIQVDQRNEAVLLPIYGSMVPFHVSTVKSVTSHQD-NRTCTIRIFFNVP   591
Os-SPT16      DV---PYARELVIQVDQKNEAVLLPIYGSMVPFHVSTVKSVTSHQD-NRTCTIRIFFNVP   589
Sb-SPT16      DV---PFVRDLVIQVDQKNEAVLLPIYGSMVPFHVSTVKSVTSHQD-NRTCTIRIFFNVP   588
Zm-Spt16      DV---PFVRDLVIQVDQKNEAVLLPIYGSMVPFHVSTVKSVTSHQD-NRTCTIRIFFNVP   601
Gm-SPT16      DL---PPPREMMIQIDQKSEAVLLPINGSMVPFHVAFIRTVSSQQDTNRNCYIRIIFNVP   599
Pt-SPT16      DI---PPARDLMIQIDQKNEAVLLPIYGNMVPFHVSTIRTVSSQQDTNRTCYIRIIFNVP   599
Vv-SPT16      DL---PPPKELMIQVDQKNEAILLPIYGSMVPFHVATVKSVSSQQDTNRTCYIRIIFNVP   603
Sm-SPT16      EL---PFSRELMIQVDQKNEAVLLPIYGIMVPFHIATVRTINNHQDL-NSSIIRIIFNVP   597
Pp-SPT16      DI---PA-RELKIHVDQKNEAVLLPVYGLLVPFHIATVKSVSSQQDG-GHSYIRIIFNVP   594
Hs-SPT16      LMPKEPHIREMKIYIDKKYETVIMPVFGIATPFHIATIKNISMSVEG-DYTYLRINFYCP   575
Dm-SPT16      QMPREPEVKELKLYVDKKYETVIMPVFGIQVPFHISTIKNISQSVEG-EYTYLRINFFHP   581
               :      ::: : :* : :::::*: *  .***:   :. .   :      :*: *  *

Sc-SPT16      GSSGGISKKVEELPYEESADNQFVRSITLRSKDGDRMSET----------FKQIADLKKE   649
At-SPT16      GTPFNPH----DSNSLKNQGAIYLKEVSFRTKDSRHSSEV----------TQQIKTLRRQ   648
Ta-SPT16      GMPFSND------KDLKSQGAIYLKEITFRSKDPRHSSEV----------VQQIKTLRRQ   635
Os-SPT16      GMPFSND------SNLKSQGAIYLKEITFRSKDPRHSSEV----------VQQIKTLRRQ   633
Sb-SPT16      GMPFSND------SKLNSQGAIYLKEITFRSKDPRHSSEV----------VQQIKTLRRQ   632
Zm-Spt16      GMPFSND------SKFNSQGAIYLKEITFRSKDPRHSSEV----------VQQIKTLRRQ   645
Gm-SPT16      GTPFSPH----DSNSMKFQGSIYLKEASFRSKDSRHISEV----------VQSIKTLRRQ   645
Pt-SPT16      GAAFNPH----DSNSLKHQGAIYLKEVSFRSKDPRHISEV----------VQLIKTLRRH   645
Vv-SPT16      GTPFSPH----DSNSMKFQGSIYLKEVSFRSKDPRHISEV----------VQMIKTLRRQ   649
Sm-SPT16      GAGFTTN----DVPFQKFPHMIYLKEISFRTSDIKHSTQI----------VQMMKTLKRQ   643
Pp-SPT16      GAGFGPN----DVPTQKFPRSIYVKEVSFRSNDTRHSYQV----------VQLIKTLRRQ   640
Hs-SPT16      GSALGRN----EGNIFPNPEATFVKEITYRASNIKAPGEQTVPALNLQNAFRIIKEVQKR   631
Dm-SPT16      GATMGRN----EGGLYPQPEATFVKEVTYRSSNVKEHGEVGAPSANLNNAFRLIKEVQKR   637
              *                      :::. : *:.:     :            : :  :::.

Sc-SPT16      ATKREQERKALADVVQQDKLIENK-TGRTKRLDQIFVRPNPDT--KRVPSTVFIHENGIR   706
At-SPT16      VMARESERAERATLVTQEKLQLAGNKFKPLRLSELWIRPPFSGR-KKIPGTLEAHANGFR   707
Ta-SPT16      VASRESERAERATLVTQEKLQQASNKTKQMRLNDVWIRPPFGGRGRKLTGTLEAHVNGFR   695
Os-SPT16      VASRESERAERATLVTQEKLQLTSNRNKPVRLSDVWIRPAFGGRGRKLTGTLESHVNGFR   693
Sb-SPT16      VASRESERAERATLVTQEKLQIGNNRMKMMRLSDVWIRPAFGGRGRKLTGNLEAHFNGFR   692
Zm-Spt16      VASRESERAERATLVTQEKLQIGSNRMKMMRLSDVWIRPAFGGRGRKLTGNLEAHFNGFR   705
Gm-SPT16      VVARESERAERATLVTQEKLQLANNRFKPIRLSDLWIRPAFGGRGRKIPGTLEAHVNGFR   705
Pt-SPT16      VVARESERAERATLVTQEKLQLAGNRFKPIRLTDLWIRPVFTGRGRKLPGALEAHVNGFR   705
Vv-SPT16      VASRESERAERATLVTQEKLQLAGTRFKPIRLSDLWIRPSFGGRGRKLTGSLESHTNGFR   709
Sm-SPT16      VSQRESEKAERATLVTQEKLQISK--GKAIRLSDLWIRPPFAGRKRR-RGTLEAHVNGLR   700
Pp-SPT16      VAQRESERAERATLVTQERLQIGK--GRPIRLSDLWIRPAFGGRGRKMSGTLEAHTNGFR   698
Hs-SPT16      YKTREAEEKEKEGIVKQDSLVINLNRSNP-KLKDLYIRPNIAQ--KRMQGSLEAHVNGFR   688
Dm-SPT16      FKTREAEERERKEDLVKQDTLILSQNKGNP-KLKDLYIRPNIVT--KRMTGSLEAHSNGFR   694
                 ** *.      :* *: *        .   :* ::::**      ::   . :   * **:*

Sc-SPT16      FQSPLRTDSRIDILFSNIKNLIFQSCKGELIVVIHIHLKNPILMGKKKIQDVQFYREASD   766
At-SPT16      YST-TRPDERVDVLFANIKHAFFQPAEKEMITLLHFHLHNHIMVGTKKTKDVQFYVEVMD   766
Ta-SPT16      YST-SRADERVDIMYGNIKHAFFQPAEKEMITLLHFHLHNHIMVGNKKTKDVQFYVEVMD   754
Os-SPT16      YST-SRADERVDIMYGNVKHAFFQPAEKEMITLLHFHLHNHIMVGNKKTKDVQFYVEVMD   752
Sb-SPT16      YST-SRSDERVDIMFGNIKHAFFQPAEKEMITLLHFHLHNHIMVGNKKTKDVQFYVEVMD   751
Zm-Spt16      YST-SRSDERVDIMFGNIKHAFFQPAEKEMITLLHFHLHNHIMVGNKKTKDVQFYVEVMD   764
Gm-SPT16      YST-TRQDERVDIMFGNIKHSFFQPAENEMITLLHFHLHNHIMVGNKKTKDVQFYVEVMD   764
Pt-SPT16      FST-SRSEERVDIMFSNIKHAFFQPAEKEMITLLHFHLHNHIMVGNKKTKDVQFYVEVMD   764
Vv-SPT16      YST-SRPDERVDIMYGNIKHAFFQPAEKEMITLLHFHLHNHIMVGNKKTKDVQFFVEVMD   768
Sm-SPT16      YST-MKAEETVDILYRNIRHAFFQPAEKEMITLLHFHLHNHIMVGNKKAKDVQFFVEVMD   759
Pp-SPT16      YST-MRQEEKVDIMYRNIKHAFFQPAEKEMITLVHFHLHNYIMVGTKKTKDVQFYVEVME   757
Hs-SPT16      FTS-VRG-DKVDILYNNIKHALFQPCDGEMIIVLHFHLKNAIMFGKKRHTDVQFYTEVGE   746
Dm-SPT16      YIS-VRG-DKVDILYNNIKSAFFQPCDGEMIILLHFHLKYAIMFGKKKHVDVQFYTEVGE   752
              : :  :   . :*::: *::  :**  .. *:* ::*:**:  *:.*.*:  ****: *. :

Sc-SPT16      MSVDETGGGRRGQSRFRRYGDEDELEQEQEERRKRAALDKEFKYFADAIAEA------SN   820
At-SPT16      VVQSL-GGGRR------SAYDPDEIDEEQRERDRKNKINMDFNHFANRVNDMWQLPQFAS   819
Ta-SPT16      VVQTV-GGSRR------SALDPDEIEEEQRERDRKNRINMEFQNYINKVNDHWSQPQFKG   807
Os-SPT16      VVQTL-GGNRR------SALDPDEIEEEQRERDRKNRINMDFQNFVNKVNDHWSQPQFKG   805
Sb-SPT16      VVQTL-GGSRR------SALDPDEIEEEQRERDRKNRINMDFQNFVNKVNDHWSQPQFKG   804
Zm-Spt16      VVQTL-GGSRR------SALDPDEIEEEQRERDRKNRINMDFQNFVNKVNDHWSQPQFKG   817
Gm-SPT16      MVQNV-GGGKR------SAYDPDELEEEQRERDRKNKINVEFQTFVNRLNDLWGQPQFNG   817
Pt-SPT16      VVQTL-GGGKR------SAYDPDEIEEEQRERDRKNKINMDFQSFVNRVNDLWSQPQFSG   817
Vv-SPT16      VVQTL-GGGKR------SAYDPDEIEEEQRERDRKNKINMDFQNFVNRVNDLWGQPQFKG   821
Sm-SPT16      GVQNV-GGSRR------SHFDPDEIEEEQAERERKNKLNKEFEVFVKKVTDLWEQPALRN   812
Pp-SPT16      VVQTL-GGSRR------SMMDPDEIEEEQQERDRRNKINKEFEAFVKRMAELWDQPPWRE   810
Hs-SPT16      ITTDL-GKH-Q------HMHDRDDLYAEQMEREMRHKLKTAFKNFIEKVEAL------TK   792
Dm-SPT16      ITTDL-GKH-Q------HMHDRDDLAAEQAERELRHKLKTAFKSFCEKVETM------TK   798
                    *   :         * *::  ** **  :  :.  *: : . :
```

```
Sc-SPT16    GLLTVENTFRDLGFQGVPNRSAVFCMPTTDCLVQLIEPPFLVINLEEVEICILERVQFGL   880
At-SPT16    LDLEFDQPLRELGFHGVPHKTSAFIIPTSSCLVELIEYPFLVVSLSEIEIVNLERVGFGQ   879
Ta-SPT16    LDLEFDIPLRELGFHGVPYKASAFIIPTSTCLVELIETPFLVVTLGEIEIVNLERVGFGT   867
Os-SPT16    LDLEFDVPLRELGFHGVPYKASAFIIPTSTCLVELIETPFLVVTLSEIEIVNLERVGFGT   865
Sb-SPT16    LDLEFDVPLRELGFYGVPYKASAFIIPTSTCLVELIENPFLVVSLSEIEIVNLERVGFGT   864
Zm-Spt16    LDLEFDVPLRELGFHGVPYKASAFIIPTSTCLVELIETPFLVVSLSEIEIVNLERVGFGT   877
Gm-SPT16    LDLEFDQPLRELGFPGVPHKSSVFIVPTSACLVELIETPFLVVTLSEIEIVNLERVGLGQ   877
Pt-SPT16    LDLEFDQPLRELGFHGVPHKVTSFIVPTSSCLVELVETPFLVVTLGEIEIVNLERVGLGQ   877
Vv-SPT16    LDLEFDQPLRELGFHGVPHKASAFIVPTSSCLVELIETPFLVITLSEIEIVNLERVGLGQ   881
Sm-SPT16    YGLEFDIPFRELGFHGVPNKTSAFIVPTVKCLVELIEFPFLVVTVEDIELVNLERVGFAQ   872
Pp-SPT16    LDLEFDIPFRELGFHGVPNKSSAFIVPTVNCLVELIETPFLVVSLNDIEIVNLERVGLGQ   870
Hs-SPT16    EELEFEVPFRDLGFNGAPYRSTCLLQPTSSALVNATEWPPFVVTLDEVELIHFERVQFHL   852
Dm-SPT16    SVVEFDTPFRELGFPGAPFRSTVTLQPTSGSLVNLTEWPPFVITLDDVELVHFERVQFHL   858
              : .:  :*:*** *.* : :    **  .**:  * * :*:.: ::*:  :*** :

Sc-SPT16    KNFDMVFVYKDFNKPVTHINTVPIESLDFLKQWLTDMDIPYTVSTINLNWATIMKSLQDD   940
At-SPT16    KNFDMAIIFKDFKKDVLRVDSVPTSSLEGIKEWLDTTDIKYYESKLNLNWRQILKTITDD   939
Ta-SPT16    KNFDMAIVFKDFKKDVLRIDSIPSTSLDAIKEWLDTTDLKYYESRLNLNWRPILKTIIDD   927
Os-SPT16    KNFDMAIVFKDFKKDVLRIDSIPSTSLDAIKEWLDTTDLKYYESRLNLNWRPILKTIIDD   925
Sb-SPT16    KNFDMAIVFKDFKKDVLRIDSIPSASLDAIKEWLDTTDLKYYESRLNLNWRPILKTIIDD   924
Zm-Spt16    KNFDMAIVFKDFKKDVLRIDSIPSASLDAIKEWLDTTDLKYYESRLNLNWRPILKTIIDD   937
Gm-SPT16    KNFDMTVVFKDFKRDVLRIDSIPSTSLDGIKEWLDTTDIKYYESRLNLNWRQILKTITDD   937
Pt-SPT16    KNFDMTIVFKDFKRDVLRIDSIPSTSLDGIKEWLDTTDIKYYESRLNLNWRQILKTITDD   937
Vv-SPT16    KNFDMTIVFKDFKRDVLRIDSIPSTSLDGIKEWLDTTDLKYYESRLNLNWRPILKTITED   941
Sm-SPT16    KAFDMAIIFKDFKKDVLRIDAIPSTSLDNIKEWLNSMAIKYYESRMNLNWRPILKTILDD   932
Pp-SPT16    KAFDMAIVFKDFKREVLRIDAIPSTSLDGIKEWLNSMNIKYYESRMNLNWRPILKTILED   930
Hs-SPT16    KNFDMVIVYKDYSKKVTMINAIPVASLDPIKEWLNSCDLKYTEGVQSLNWTKIMKTIVDD   912
Dm-SPT16    RNFDMIFVFKEYNKKVAMVNAIPMNMLDHVKEWLNSCDIRYSEGVQSLNWQKIMKTITDD   918
            : *** .::*::.: *  ::::*   *: :*:**    : *  .  .***  *:*:: :*

Sc-SPT16    PYQFFLDGGWNFLATGSDDEASD----ESEEEVSEYEASEDDVSDESAFSEDEEGSEVDD   996
At-SPT16    PQSFIDDGGWEFLNLDGSDSESG----GSEESDKGYEPSDVEVESESEDEASESESLVES   995
Ta-SPT16    PQKFVDDGGWEFLNMEASDSEAE----ETEESDQGYEPSDAEPESESEEEDSDSASLVES   983
Os-SPT16    PQKFIDDGGWEFLNMEASDSETE----ETEESDQGYEPSDAEPESESEDEDSDSESLVES   981
Sb-SPT16    PQKFIDDGGWEFLNMEASDSETE----DTEESDQGYVPSDAEPESESEDDDSDSESLVES   980
Zm-Spt16    PQKFIDDGGWEFLNMEASDSETE----DTEESDQGYVPSDAEPESESEDDDSDSESLVES   993
Gm-SPT16    PQSFIEGGGWEFLNLEATDSESE----NSEESDKGYEPSDVEPESDSEDEASDSESLVES   993
Pt-SPT16    PQSFIDDGGWEFLNLEASDSDSD----NSEDSDQGYIPSDAEPESESEDDVSDSESLVES   993
Vv-SPT16    PEKFIEDGGWEFLNLEVSDSDSE----NSQESDQGYEPSDVQPSDTGSEEEGDDSESLVES   997
Sm-SPT16    PKKFIDDGGWEFLNMEASDSESE----KSEESDKGYEPSDLEEPSESEDEGSDDESVVES   988
Pp-SPT16    PDKFIEDGGWEFLNMEASDSESD----KSEESDEGYEPSDVEVVSESEDDDSDDESVVES   986
Hs-SPT16    PEGFFEQGGWSFLEPEGEGSDAEEGDSESEIEDETFNPSEDDYEEEEED--SDEDYSSEA   970
Dm-SPT16    PEGFFEQGGWTFLDPESGSEGENE--TAESEEDEAYNPTDAES-DEESD--EDSEYSEAS   973
            *  *.  *** **     ..         . . . :  :: :    .    .:.

Sc-SPT16    DISGDESEDYTGDESEEGEDWDELEKKAARADRGANFRD---------------------   1035
At-SPT16    -DDDEEEDSEQESEEEKGKTWDELEREATNADREHGVESDSEEERKRRKMKAFGKSRPGT   1054
Ta-SPT16    -DEDEEEDSDEDSEEEKGKTWDELEREATNADRDHGAESDSEEERRRRKVKTFSKSGAPP   1042
Os-SPT16    -DEDDEDDSEEDSEEEKGKTWEELEREASNADREHGAESDSEEERRRRKVKTFSKSRPPP   1040
Sb-SPT16    -DDDDE-ESDEDSEEEKGKTWEELEREASNADREHGAESDSEEERRRRKAKTFSKSRAPE   1038
Zm-Spt16    -DDDDE-ESDEDSEEEKGKTWEELEREASNADREHGAESDSEEERRRRKAKTFGKSRAPE   1051
Gm-SPT16    -EDDGEEDSEEDSEEEKGKTWEELEREASNADREKGNESDSEEDRKRRKAKSFGKSRGAG   1052
Pt-SPT16    -EDDEEEEDEEDSEEEKGKTWEELEREASNADREKGDDSDSEEERNRRKVKTFGKSRPAP   1052
Vv-SPT16    -EDDVEEDSDGDSEEEQGKTWEELEREASNADREKGDESDSEEERKRRKMKAFGKARVPE   1056
Sm-SPT16    -EDDEE--EEADSDEEEGMSWDELEAKASKEDKEKGDESDSEDERRRRKAKMTGKTRASP   1045
Pp-SPT16    -DDDEA-EEEEDSEEEEGLTWDQLEEAAKRDDKMKGDEEDSEDERHRNRKKAAGKGRMPD   1044
Hs-SPT16    -EESDYSKESLGSEEESGKDWDELEEEARKADRESRYEEEEEQSRSMSRKR---KASVHS   1026
Dm-SPT16    -EDSEESDEDLGSDEESGKDWSDLEREAAEEDRNHDYAADDKPRNGKFDSKKHGKSSKHS   1032
              ..    .   .:.*.*  *.:**  * . *:

Sc-SPT16    -------------------------------------------------------        1035
At-SPT16    SGGGGSSSMKN-----------------M--PPSK----------------RKHR        1074
Ta-SPT16    QRGPSSSKHAPLPQRGG----SSSGNKSR--PPPSSSK------GGPPSKKPKFR        1085
Os-SPT16    ER--------------------------------SSFK------G-GPSKKPKFR        1056
Sb-SPT16    -R--------------------------------SSFK------GAPPSKKPKFR        1054
Zm-Spt16    -R--------------------------------SSFK------GAPPSKKPKFR        1067
Gm-SPT16    L------------------------------------S-------SSMTKRPKLR        1064
Pt-SPT16    RPAPRPAPR----------------PAPR--PPPRTVP-------GSMPKRPKFR        1082
Vv-SPT16    ---------------------------------KRSTR-------GSLPKRPKLR        1071
Sm-SPT16    K--------------------------------------------APPAKRFKTR        1056
Pp-SPT16    PR---------------------------------DAK------RGQPNKRPKVR        1060
Hs-SPT16    SGRG--------SNRGSRHSSA---------PPKKKR------------------        1047
Dm-SPT16    PSKSSK---DKYNSRDKHHSSSSSGNKSSSKDKDRKRSRDDSRDNGHKSKKSRH-        1083
```

Figure S2. Amino acid sequence alignment of SPT16 from different organisms. Sequences (plant, green; metazoan, red; fungal, blue) were retrieved by database searches using blastp (https://blast.ncbi.nlm.nih.gov/) using At-SPT16 as query. The sequences were aligned using Clustal Omega (https://www.ebi.ac.uk/Tools/msa/clustalo/). The species of which the protein sequences were retrieved are indicated as follows: *Arabidopsis thaliana (At), Drosophila melanogaster (Dm), Glycine max (Gm), Homo sapiens (Hs), Oryza sativa (Os), Physcomitrella patens (Pp), Populus trichocarpa (Pt), Saccharomyces cerevisiae (Sc), Selaginella moellendorfii (Sm), Sorghum bicolor (Sb), Triticum aestivum (Ta), Vitis vinifera (Vv), Zea mays (Zm).*
